# RFWD3 modulates response to platinum chemotherapy and promotes cancer associated phenotypes in high grade serous ovarian cancer

**DOI:** 10.3389/fonc.2024.1389472

**Published:** 2024-04-22

**Authors:** Sarah J. Taylor, Robert L. Hollis, Charlie Gourley, C. Simon Herrington, Simon P. Langdon, Mark J. Arends

**Affiliations:** ^1^ Edinburgh Pathology, Cancer Research UK Scotland Centre, Institute of Genetics and Cancer, University of Edinburgh, Edinburgh, United Kingdom; ^2^ Nicola Murray Centre for Ovarian Cancer Research, Cancer Research UK Scotland Centre, Institute of Genetics and Cancer, University of Edinburgh, Edinburgh, United Kingdom

**Keywords:** ovarian cancer, RFWD3, DNA repair, proliferation, migration

## Abstract

**Background:**

DNA damage repair is frequently dysregulated in high grade serous ovarian cancer (HGSOC), which can lead to changes in chemosensitivity and other phenotypic differences in tumours. RFWD3, a key component of multiple DNA repair and maintenance pathways, was investigated to characterise its impact in HGSOC.

**Methods:**

RFWD3 expression and association with clinical features was assessed using *in silico* analysis in the TCGA HGSOC dataset, and in a further cohort of HGSOC tumours stained for RFWD3 using immunohistochemistry. RFWD3 expression was modulated in cell lines using siRNA and CRISPR/cas9 gene editing, and cells were characterised using cytotoxicity and proliferation assays, flow cytometry, and live cell microscopy.

**Results:**

Expression of RFWD3 RNA and protein varied in HGSOCs. In cell lines, reduction of RFWD3 expression led to increased sensitivity to interstrand crosslinking (ICL) inducing agents mitomycin C and carboplatin. RFWD3 also demonstrated further functionality outside its role in DNA damage repair, with RFWD3 deficient cells displaying cell cycle dysregulation, reduced cellular proliferation and reduced migration. In tumours, low RFWD3 expression was associated with increased tumour mutational burden, and complete response to platinum chemotherapy.

**Conclusion:**

RFWD3 expression varies in HGSOCs, which can lead to functional effects at both the cellular and tumour levels.

## 1 Introduction

Ovarian cancer is the eighth most common cancer in women, with over 300, 000 new diagnoses each year (1). Of these, high grade serous ovarian cancer (HGSOC) is the most prevalent subtype, accounting for approximately 70% of cases (2). HGSOC is frequently diagnosed at advanced stages with extensive local and distant metastases, and carries a poor prognosis, with five-year survival rates of just 40% (3). One of the major challenges in the management of HGSOC, and a contributor to the high mortality rates, is the development of resistance to standard of care platinum-based chemotherapy despite initial chemosensitivity (4).

HGSOC is characterized by a lack of frequent mutations, except in *TP53*, which occur in 97% of cases, and the *BRCA1/2* genes which are collectively mutated in 22% of cases (5). Aside from these, HGSOCs are largely driven by copy number alterations and exhibit high levels of genomic instability (5). The BRCA1/2 proteins function in the homologous recombination (HR) mediated repair of DNA double-strand breaks (DSBs), and are a key component of the Fanconi anaemia (FA) pathway which repairs DNA interstrand crosslinks (ICLs) induced by platinum treatment (6). The impact of *BRCA1/2* mutation in HGSOCs has been extensively studied, with BRCA1/2 deficient tumours associated with a highly platinum sensitive phenotype and favourable patient prognosis (7–9). Homozygous (or compound heterozygous) mutation of any of the essential FA genes involved in the FA pathway causes Fanconi anaemia, a developmental disorder underpinned by genomic instability and the inability to repair ICLs (10). However, non-BRCA members of the FA pathway remain less well studied both in HGSOC and other cancer types (11).

RING finger and WD repeat domain-containing protein 3 (RFWD3) (aka FANCW) is an E3 ligase which was first identified as a regulator of cell cycle checkpoint control (12). More recent work has shown that, like BRCA1/2, RFWD3 is essential for repair of ICLs via the FA pathway (13), functioning both in HR repair of DSBs and gap filling translesion synthesis (TLS) across DNA lesions (14, 15). RFWD3 exhibits promiscuous ubiquitin ligase activity, targeting multiple substrates involved in the DNA damage response and maintenance of genomic stability, including cell cycle checkpoint maintenance, replication fork stability, and DNA replication (16–18).

Studies of RFWD3 in HGSOC have not been reported in the literature, and investigation in other cancer types has been limited. It has however, been reported as a potential tumour promoter in gastric, colorectal, pancreatic, and non-small cell lung cancers (19–22).

In this study, we therefore aimed to investigate the role of RFWD3 in HGSOC, by evaluating links between RFWD3 expression and clinical characteristics in HGSOC patient samples, and defining the function of RFWD3 in HGSOC cell line models.

## 2 Methods

### 2.1 The Cancer Genome Atlas high grade serous ovarian cancer dataset

*In silico* analyses of *RFWD3* expression were carried out using The Cancer Genome Atlas (TCGA) dataset of 489 HGSOC cases (TCGA-OV) described in (5). Median follow up was 30 months (range 0-179 months). In this cohort, overall survival was defined as the interval from initial surgical resection to the last known contact or death. Disease free survival was defined as the interval from initial surgical resection to disease progression, date of recurrence, or date of last known contact if the patient was alive and had not recurred. Analysis was performed using the cBioportal online tool (www.cbioportal.org) (23) and Graphpad Prism v9.4.1.

### 2.2 Cell culture

Human HGSOC cell lines ES2, 59M, COV318 and PEO1 were obtained from in-house liquid nitrogen frozen stocks. Cell lines were authenticated by short tandem repeat profiling, and regularly mycoplasma tested. Cells were grown in RPMI-1640 (Thermo Fisher, Loughborough, UK) supplemented with 10% fetal bovine serum (Thermo Fisher), 1% penicillin streptomycin (Thermo Fisher), and 2mM L-glutamine (Thermo Fisher).

### 2.3 Western blot analysis of protein expression

Cultured cells were treated with lysis buffer on ice, centrifuged at 15,000g, and supernatant collected. Protein content was quantified using the bicinchoninic acid assay. Lysates were loaded on 7.5% acrylamide gels, and subjected to SDS-PAGE to separate proteins. Proteins were transferred to PVDF membrane, incubated with blocking buffer (LI-COR, Cambridge, UK) for 1h, and incubated overnight with primary antibody diluted in blocking buffer. Primary antibodies targeting RFWD3 (Abcam, Cambridge, UK; ab138030; 1:500 dilution), tubulin (Abcam; ab7291; 1:5000 dilution), PARP (Cell Signaling Technology, Danvers, MA, USA; 9542; 1:1000 dilution) and FANCD2 (Abcam; ab108928; 1:1000 dilution) were used. Membranes were incubated with anti-mouse IRDye 680LT (LI-COR 926-68020) and anti-rabbit IRDye 800CW (LI-COR 926-68023), and imaged using the Odyssey Infrared Imaging System (LI-COR Biosciences). Quantification of band intensity was performed using ImageJ v1.53 (24).

### 2.4 Sulforhodamine B assay

Cytotoxicity and proliferation were measured using the SRB assay. 250-3000 cells were plated per well in 96-well plates, treated with 1:4 or 1:2 dilution series of carboplatin (Sigma-Aldrich, Gillingham, UK), mitomycin C (Selleckchem, Houston, TX, USA) paclitaxel (Selleckchem), or olaparib (Selleckchem) and incubated for 5 days at 37°C. These were fixed using 25% trichloroacetic acid (Sigma-Aldrich), and stained with 0.4% w/v sulforhodamine B dye (Sigma-Aldrich). Dye was dissolved by addition of Tris pH 10.5, and optical density at 540nM was measured using a BP800 Microplate Reader (Biohit). Absolute IC_50_ values were interpolated from concentration-response curves using Graphpad Prism v9.4.1.

### 2.5 Transient siRNA transfection of cell lines

Transfection of cell lines with siRNA was performed using Lipofectamine 3000 (Thermo Fisher) according to manufacturer’s instructions. *RFWD3* targeting siRNA CCAUUUGAGGUGAACCGUAtt or negative control siRNA (1027281, Qiagen, Manchester, UK) was used. Transfection was performed twice, with cells allowed to recover for 24h between transfections.

### 2.6 CRISPR/cas9 mediated gene editing

sgRNA sequences targeting two distinct sequences in the *RFWD3* gene (CACCGCTCTCAGGGTTCGGGCATAA and CACCGGGCTCTCAGCATTACGCTGT; Integrated DNA technologies (IDT), Ilkeston, UK) were cloned into pSpCas9(BB)-2A-Puro (PX459) V2.0 plasmid backbones (25). Cells were transfected with the resulting plasmids using Lipofectamine 3000 (Thermofisher) according to manufacturer’s instructions. Selection of transfected cells was carried out for 48h using 2.5µg/mL puromycin, and individual cells were subcloned to generate monoclonal cell lines. Monoclonal cell lines were evaluated for gene editing via Western blot and Sanger sequencing. CRISPR/cas9 mediated editing was carried out using the same methodologies as described for *RFWD3*, and sgRNA sequences targeting two positions in the *FANCD2* gene (CACCGCATCCTCAATGTAAGACTCC and CACCGGATAGGAAGGGTGTCTCCTC; IDT).

### 2.7 Sanger sequencing of CRISPR/cas9 edited cell lines

DNA extractions were carried out using an Allprep DNA/RNA kit (Qiagen) following manufacturer’s instructions. DNA was amplified for sequencing via PCR using AmpliTaq Gold 360 PCR Master Mix (Thermo Fisher) and following manufacturers’ protocols. PCR cycling conditions were as follows; 95˚C for 5 minutes, followed by 40 cycles (95˚C for 30 seconds, 55˚C for 30 seconds, and 72˚C for 30 seconds), and 72˚C for 5 minutes. Forward primer sequence GTTCTGTAGCCCTTTTGATTGTATCAT, reverse primer sequence TTTCTGTATGGAGAACTGCTGTGG (IDT). PCR product was analysed for heterozygosity by gel electrophoresis and Sanger sequenced.

### 2.8 Cell cycle analysis

Cells were seeded in 10cm dishes, and treated with 10µM bromodeoxyuridine (BrdU; Abcam) for 30min at 37°C. 2x10^6^ cells were fixed in 70% (v/v) ethanol. These were digested to nuclei using 1mg/mL pepsin (Sigma-Aldrich), treated with 2M hydrochloric acid, and blocked using 0.5% BSA (Sigma-Aldrich) solution. Staining was with 1:100 anti-BrdU antibody (Invitrogen; 14-5071-82), followed by anti-mouse AF488 secondary antibody (A11029, Invitrogen). Nuclei were then treated with RNase A (Thermofisher) and 50µg/mL propidium iodide (PI; Thermofisher), and analysed using the LSRFortessa (Becton Dickinson, Franklin Lakes, USA). Cell cycle phase was assigned based on PI and BrdU staining gates in FlowJo v10.8.1.

### 2.9 Cell proliferation analysis

A total of 3000 cells were seeded per well, and were fixed and stained as described above using the SRB assay. Plates were fixed at D0, 6h, 24h, 48h, and 72h time points. 6 wells were seeded per condition, and the mean absorbance was used to plot growth curves. Doubling times were calculated from growth curves plotted in Graphpad Prism v9.4.1.

### 2.10 Cell migration analysis

Cell migration was analysed using the Incucyte S3 live imaging system (Essen, Royston, UK). 96 well Imagelock plates (Essen) were coated with 50µg/mL collagen I (prepared in-house according to (26)) or 10µg/mL laminin (Thermo Fisher). 1000 cells were seeded per well, and imaged every 15 min. Image sequences were analysed using the mTrackJ plugin on ImageJ v1.53. Individual cells were tracked for 10h, and mean migration velocity per track was calculated using mTrackJ (27). 45 cells were tracked per biological replicate.

### 2.11 Tumour microarray

Microarrays of HGSOC patient tumours are described in (28). Briefly, from ovarian cancer cases treated at the Edinburgh Cancer Centre prior to 2007, 365 high grade serous ovarian cancer cases were identified by expert pathological review. All were treated with first line platinum containing chemotherapy after primary debulking surgery. Samples of treatment naïve tumour were taken at primary surgery, fixed in formalin and embedded in paraffin. Sections were cut for immunohistochemistry (IHC) staining. Median follow up was 13.5 years (range 0.2-22.7 years). Overall survival (OS) in this cohort was defined as the interval from pathologically confirmed diagnosis to patient death. Progression free survival (PFS) was defined as the interval from pathologically confirmed diagnosis to disease progression or recurrence, respectively. Ethical approval was obtained from South East Scotland Human Annotated Bioresource (Lothian NRS Bioresource Ethics Committee reference 20/ES/0061-SR705 and SR1518). This study was carried out in accordance with the principles of the Declaration of Helsinki.

### 2.12 Immunohistochemistry staining

Slides were dewaxed and rehydrated using the Leica Autostainer XL (Leica, Newcastle, UK), and treated with 3% hydrogen peroxide solution (Sigma-Aldrich). Antigen retrieval was performed by heating slides in a pressure cooker containing Tris-EDTA buffer for 12 min. Slides were permeabilised using 0.5% Triton-X100. Blocking was with 5% goat serum for 1h. Antibody staining was with anti-RFWD3 antibody (Proteintech, Manchester, UK) (19893-1-AP) diluted 1:400 overnight at 4°C. Slides were then incubated with peroxidase conjugated secondary antibody (A0545 Sigma-Aldrich) and 3, 3’-diaminobenzidine (DAB) solution (Agilent, Wokingham, UK) for 10 min. Slides were then dehydrated and counterstained with haematoxylin on the Leica Autostainer XL before mounting. Slides were imaged in the brightfield channel using the Nanozoomer XR (Hammamatsu, Welwyn Garden City, UK).

### 2.13 Tumour microarray analysis

IHC staining of the tumour microarray (TMA) was analysed using QuPath software version 0.3.2 (https://qupath.github.io/) (29). Cells were automatically detected, and tumour areas annotated. Thresholds for nuclear DAB staining were set at optical density (OD) 0.1, 0.3 and 0.5 to determine negative, weak, moderate and strong staining in individual cells. Histoscores for each tumour core were generated based on the proportion of cells classified within each of these categories automatically within QuPath. Evaluable tumour cores were available for 231/365 cases. Where available, triplicate (54/231) and duplicate (84/231) cores for each case were analysed, and mean values were used for statistical analysis. Duplicate and triplicate cores demonstrated good concordance between histoscores (median coefficient of variation 12.9%).

### 2.14 Statistical analysis

All graphs were plotted using Graphpad Prism v9.4.1. The Cox proportional hazards test for performing multivariate analysis of survival data, and testing of proportionality of the variables, was performed using the survival package in R v4.2.2. All other statistics were performed in Graphpad Prism v9.4.1. Tests used are described in individual figure legends.

## 3 Results

### 3.1 Low expression of *RFWD3* occurs in a subset of high grade serous ovarian cancers and is associated with increased frequency of tumour mutations

The TCGA-OV dataset is a large and well characterised publicly available dataset consisting of 489 cases of HGSOC, with associated mRNA expression and clinical data (5). A subset of 316 cases also have coding exon sequences. This dataset was interrogated to determine whether alterations in *RFWD3* sequence or expression occur in HGSOC. Summary clinicopathological information for this cohort is given in **Supplementary Tables S1** and **S2**.

No instances of mutations in the *RFWD3* gene were observed in the dataset. However, low mRNA expression, defined as a Z-score of <-2 compared to tumours in the dataset with diploid *RFWD3*, occurred in 9% of cases (28/489). While two of these coincided with copy number deletions, the reason for low expression in the others is unknown. In contrast, high expression, defined as a Z-score of >2, occurred in just 1.4% (7/489) of cases. Low expression of *RFWD3* therefore occurs in a proportion of HGSOCs (**Supplementary Figure S1**).

**Figure 1:**
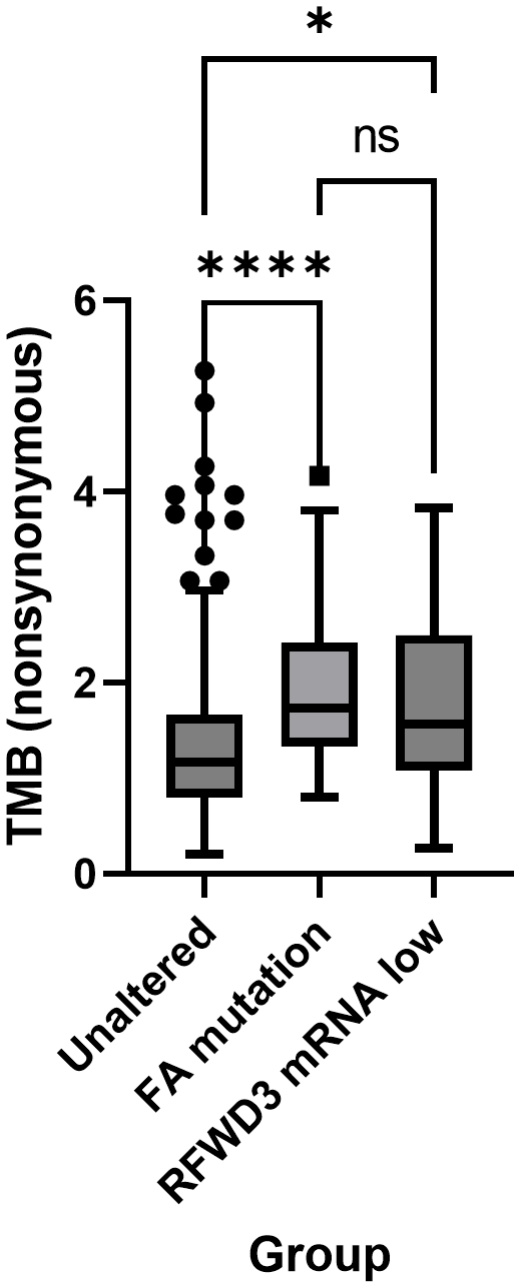
Low expression of RFWD3 is associated with increased mutation frequency in HGSOCs. Tukey’s box plot showing nonsynonymous tumour mutational burden (TMB) of tumours with low RFWD3 expression or FA gene mutations compared with other HGSOC tumours. * p<0.05, **** p<0.0001, ns, not significant, by Kruskal-Wallis test with Dunn’s multiple comparisons test. Total n=312. Control group n=207, FA mutation group n=81, RFWD3 low expression group n=24. 4 cases with both an FA mutation and low RFWD3 expression were excluded from analysis.

The subset of low *RFWD3* expressing cases were further investigated to determine whether they were associated with differences in clinical outcomes and tumour phenotype. These, and cases with mutations in other bona fide FA genes (*FANCA, FANCB, FANCC, FANCD2, FANCE, FANCF, FANCG, FANCL, FANCM, FANCI, UBE2T, SLX4, ERCC4, MAD2L2, BRIP1, BRCA2, PALB2, RAD51C, RAD51, BRCA1, XRCC2*), were compared with a control group of cases with neither an FA mutation nor low expression of *RFWD3*. There were no differences in stage or residual disease between the groups. While there was an apparent difference in patient age between the groups, no significant differences compared to the control group were observed after adjusting for multiple testing (Bonferonni corrected p=0.076) (**Supplementary Table S1**). Both the cases with low *RFWD3* expression and those with mutations in other FA genes were found to have significantly higher non-synonymous tumour mutational burden (control VS RFWD3 low expression p=0.025; control VS FA mutated p<0.0001) compared with the control group (**Figure 1**). This suggests that low *RFWD3* expression can influence tumour genotype in a similar way to mutation of FA genes including *BRCA1* and *BRCA2*.

Differences in overall survival (OS) and disease-free survival (DFS) were also assessed. While both DFS and OS were significantly improved for the FA mutated cases compared to the control group (median OS 58 VS 43 months, Bonferonni-adjusted p=0.009; median DFS 19 VS 15 months, Bonferonni-adjusted p=0.0006), there was no difference in DFS (Bonferonni-adjusted p>0.999) or OS (Bonferonni-adjusted p>0.999) between the control and RFWD3 low-expressing groups (**Supplementary Figure S2**).

### 3.2 Modulation of RFWD3 expression enhances sensitivity of high grade serous ovarian cancer cell lines to platinum

**Figure 2:**
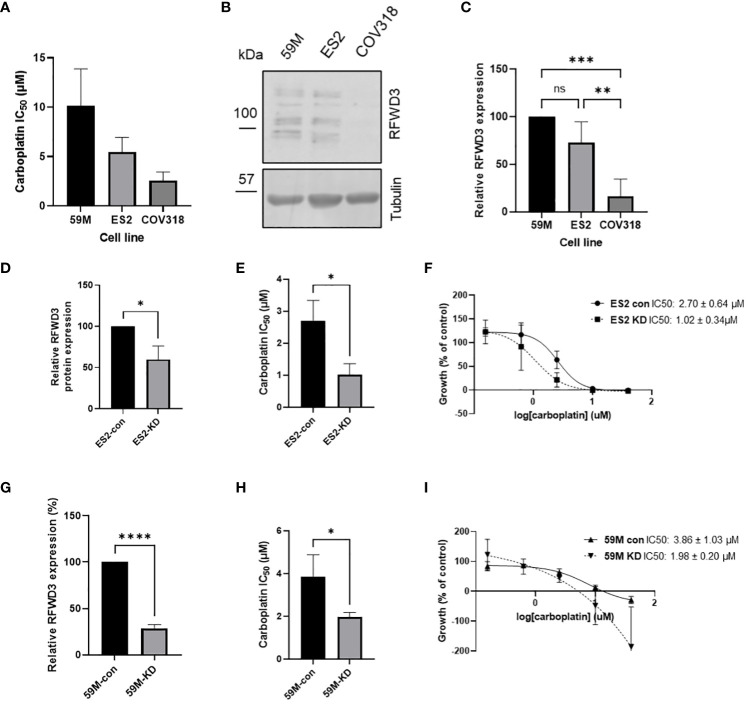
RFWD3 expression mediates carboplatin resistance in HGSOC cell lines. **(A)** IC_50_ values for carboplatin for 59M, ES2 and COV318 cell lines. Mean values of 3 biological replicates plotted. Error bars show SD. **(B)** Western blot showing RFWD3 expression in HGSOC cell lines 59M, ES2 and COV318. Full length blot shown in **Supplementary Figure S15**. **(C)** Quantification of Western blots showing expression of RFWD3 protein in 59M, ES2 and COV318 cell lines. Mean values of four biological replicates plotted. Error bars show SD. **(D)** Quantification of relative RFWD3 expression from Western blot of ES2 cells transfected with control or RFWD3 targeting siRNA, normalised to tubulin loading control and described as a percentage of control transfected cells. Error bars show SD. Mean values of 3 biological replicates plotted. **(E)** Carboplatin IC_50_ values for ES2 cell line transfected with control or RFWD3 targeting siRNA. Error bars show SD. Mean values of 3 biological replicates plotted. **(F)** Concentration response curves for ES2 cell line transfected with control or RFWD3 targeting siRNA and treated with 40 - 0.078µM carboplatin. Error bars show SD. Mean values of 3 biological replicates plotted. **(G)** Quantification of relative RFWD3 expression from Western blot of 59M cells transfected with control or RFWD3 targeting siRNA, normalised to tubulin loading control and described as a percentage of control transfected cells. Error bars show SD. Mean values of 3 biological replicates plotted. **(H)** Carboplatin IC_50_ values for 59M cell line transfected with control or RFWD3 targeting siRNA. Error bars show SD. Mean values of 3 biological replicates plotted. **(I)** Concentration response curves for 59M cell line transfected with control or RFWD3 targeting siRNA and treated with 40 - 0.078µM carboplatin. Error bars show SD. Mean values of 3 biological replicates plotted. **** p<0.0001, *** p<0.001, ** p<0.01, *p<0.05, ns, not significant. Statistics performed via ANOVA with Tukey’s multiple comparisons test **(C)** or unpaired t-test **(D, E, G, H)**.

RFWD3 expression was investigated across three HGSOC cell lines with varying carboplatin sensitivities, 59M, ES2 and COV318 (mean IC_50_ values 10.2 µM, 5.4µM, 2.6µM respectively) (**Figures 2A-C**). Although multiple bands were observed in Western blot analysis, these were all reduced in intensity by siRNA knockdown (**Supplementary Figure S3**), and likely represent different isoforms or posttranslational modifications of RFWD3. While similar RFWD3 expression was observed in two of the cell lines, the COV318 cells showed minimal expression levels (**Figure 2B**). This was significantly reduced compared with the other cell lines (p=0.0001, p=0.002 for 59M and ES2 respectively) (**Figure 2C**). Interestingly, reduced expression occurred in the most chemosensitive cell line of the three (**Figure 2A**).

Therefore, a potential link between RFWD3 expression and chemosensitivity was investigated. As the COV318, ES2 and 59M cell lines have different genetic backgrounds, and therefore a combination of multiple factors is likely responsible for differences in chemosensitivity, isogenic cell lines were used to determine whether modulation of RFWD3 could contribute to chemosensitivity. Transient knockdown of RFWD3 was performed in the 59M and ES2 cell lines using siRNA (**Supplementary Figure S3**). Endogenous expression levels of RFWD3 in the COV318 cell line were too low to obtain a measurable expression difference upon siRNA knockdown. Reduced RFWD3 expression decreased the IC_50_ of carboplatin by approximately 2-fold in both 59M (mean IC_50_ 3.9µM VS 2.0µM, p=0.036) and ES2 (mean IC_50_ 2.7µM VS 1.0µM, p=0.016) cell lines (**Figures 2D-I**). Therefore, low RFWD3 expression appeared to be associated with increased carboplatin sensitivity.

### 3.3 Generation of an RFWD3-deficient high grade serous ovarian cancer cell line

To study the effects of differential RFWD3 expression in a stable model, CRISPR/cas9 mediated gene editing was used to disrupt the *RFWD3* gene in the ES2 cell line. Two sgRNAs were designed and used to delete a DNA section of 197bp across exons 5 and 6 of the *RFWD3* gene. Clones with homozygous edits were obtained (**Supplementary Figure S4**), and the edits were confirmed by sequencing (**Supplementary Figures S5** **-** **S7**). However, edited clones still retained expression of RFWD3 protein (**Supplementary Figure S8**), although this was reduced by approximately 50% compared with empty vector transfected controls. It was therefore reasoned that the mutations may be hypomorphic, resulting in reduced protein expression. These cell lines are hereafter referred to as RFWD3^Δ/Δ^ cells. Interestingly, we were able to produce knockout cell lines using the PEO1 ovarian cancer cell line, for another FA pathway protein, FANCD2 (**Supplementary Figure S9**) using the same methodology. While multiple other groups have attempted to generate knockout models of RFWD3, these have also been unsuccessful and have resulted in similarly reduced protein levels (15, 18, 30). Therefore, *RFWD3* is speculated to be an essential gene for cell survival.

### 3.4 RFWD3^Δ/Δ^ cells show increased sensitivity to interstrand crosslink inducing agents

**Figure 3:**
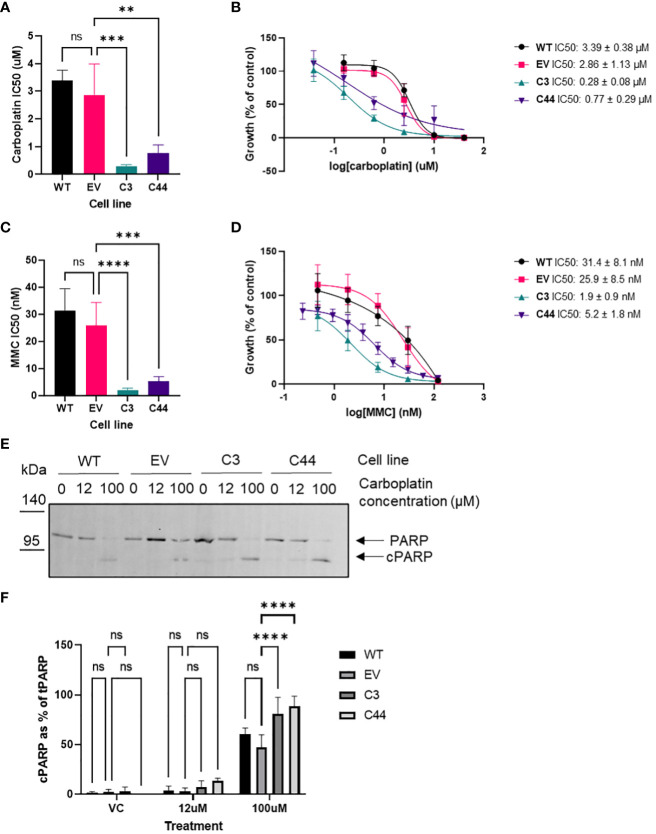
RFWD3 mediates response to ICL inducing chemotherapies. **(A)** IC_50_ values for carboplatin treated ES2 wild type, empty vector control, and RFWD3^Δ/Δ^ clones 3 and 44. Error bars show SD. Mean values of 4 biological replicates plotted. Statistical significance calculated via one way ANOVA with Dunnett’s multiple comparisons test. **(B)** Concentration response curves for ES2 wild type, empty vector control, and RFWD3^Δ/Δ^ clones 3 and 44 treated with 40 - 0.078µM carboplatin. Error bars show SD. Mean values of 4 biological replicates plotted. **(C)** IC_50_ values for mitomycin C (MMC) treated ES2 wild type, empty vector control, and RFWD3^Δ/Δ^ clones 3 and 44. Error bars show SD. Mean values of 4 biological replicates plotted. Statistical significance calculated via one way ANOVA with Dunnett’s multiple comparisons test. **(D)** Concentration response curves for ES2 wild type, empty vector control, and RFWD3^Δ/Δ^ clones 3 and 44 treated with 120 - 0.23nM mitomycin C (MMC). Error bars show SD. Mean values of 4 biological replicates plotted. **(E)** Representative Western blots of ES2 wild type, empty vector control and RFWD3^Δ/Δ^ clones 3 and 44 treated with vehicle control, 12µM or 100µM carboplatin. Upper band indicated in red represents full length PARP, lower band indicated in blue represents cPARP. Molecular weights in kDa. Full length blot shown in **Supplementary Figure S16**. **(F)** Bar chart showing cPARP normalised to total PARP for ES2 wild type, empty vector control and RFWD3^Δ/Δ^ clones 3 and 44 treated with vehicle control, 12µM or 100µM carboplatin. 4 biological replicates. Error bars show SD. Statistical significance calculated via two way ANOVA with Dunnett’s multiple comparisons test. **p<0.01, ***p<0.001, ****p<0.0001, ns, not significant.

As the siRNA data showed that changes in RFWD3 expression can modify response to platinum, the response of RFWD3^Δ/Δ^ cells to a range of chemotherapeutic drugs was assessed, in order to identify potential strategies to best treat tumours with differing expression of RFWD3. Consistent with our previous results, the RFWD3^Δ/Δ^ cells were hypersensitive to carboplatin, demonstrating an approximately 10-fold increase in sensitivity compared with controls (mean IC_50_ 2.9µM control VS 0.3µM RFWD3 ^Δ/Δ^ clone 3, p=0.0004; mean IC_50_ 2.9µM control VS 0.8µM RFWD3 ^Δ/Δ^ clone 44, p=0.003) (**Figures 3A, B**). RFWD3^Δ/Δ^ cells showed similarly enhanced sensitivity to mitomycin C (mean IC_50_ 25.9nM control VS 1.9nM RFWD3 ^Δ/Δ^ clone 3, p<0.0001; mean IC_50_ 25.9nM control VS 5.2nM RFWD3 ^Δ/Δ^ clone 44, p=0.0003) (**Figures 3C, D**); both of these drugs introduce ICLs to DNA. This validated the ability of the RFWD3^Δ/Δ^ cells to show phenotypic changes consistent with FA deficiency.

Apoptosis in response to carboplatin treatment was assessed via Western blot analysis of caspase-cleaved PARP (cPARP). The proportion of cPARP expression increased for all cell lines upon treatment with carboplatin (**Figures 3E, F**). At 100µM carboplatin treatment, there was a significant increase in the proportion of cPARP in the RFWD3^Δ/Δ^ cell lines compared with controls (mean percentage of cPARP 47% control VS 81% RFWD3 ^Δ/Δ^ clone 3, p<0.0001; mean percentage of cPARP 47% control VS 89% RFWD3 ^Δ/Δ^ clone 44, p<0.0001) (**Figure 3F**). This indicates that RFWD3 deficiency can lead to increased apoptosis upon carboplatin treatment.

Cell response to treatment with paclitaxel, another commonly utilized chemotherapeutic in HGSOC treatment, was also assessed. No consistent differences in paclitaxel sensitivity of control and RFWD3^Δ/Δ^ cells were observed (**Supplementary Figure S10**). Therefore, ICL inducing agents appear to be the most effective treatment for tumour cells with low expression of RFWD3. To assess whether there was a differential response to PARP inhibitors, the response of the RFWD3 Δ/Δ clone 3 was compared with the control clones, however only a non-significant small change was observed (**Supplementary Figure S11**).

### 3.5 RFWD3 participates in tumour relevant pathways beyond DNA damage repair

RFWD3 has been previously reported to participate in non-canonical signalling pathways distinct from its role in ICL repair in other cancer types (21, 22). Therefore, the effect of RFWD3 deficiency on cancer relevant phenotypes was assessed using the RFWD3^Δ/Δ^ cell lines.

**Figure 4:**
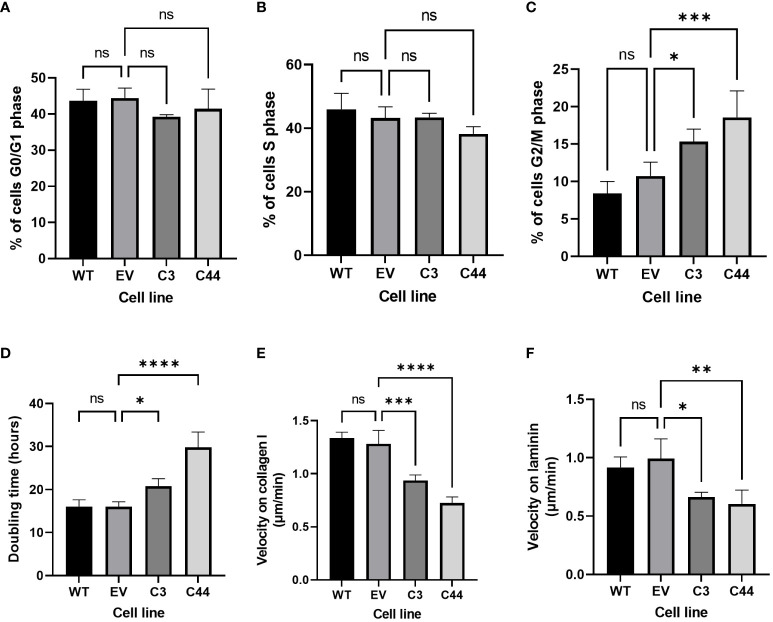
Non-canonical roles of RFWD3 in HGSOC cells. **(A-C)** Percentage of cells in **(A)** G0/G1, **(B)** S, and **(C)** G2/M phase of the cell cycles for ES2 wild type (WT), empty vector (EV) control, and RFWD3Δ/Δ clones 3 and 44 C3 and C44). 4 biological replicates. **(D)** Doubling times for ES2 wild type, empty vector control, and RFWD3Δ/Δ clones 3 and 44 in culture. 4 biological replicates. **(E, F)** Mean 2D migration velocity of for ES2 wild type, empty vector control, and RFWD3Δ/Δ clones 3 and 44 on **(E)** collagen I and **(F)** laminin. 3 biological replicates. Error bars show SD. Statistics calculated via ANOVA with Dunnett’s multiple comparisons test. *p<0.05, **p<0.01, ***p<0.001, ****p<0.0001, ns, not significant.

Proportions of cells in different phases of the cell cycle were assessed using flow cytometry (**Supplementary Figure S12**). There was no difference in the proportions of cells in G0/G1 or S-phase of the cell cycle (**Figures 4A, B**). However, both RFWD3^Δ/Δ^ clones showed an accumulation of cells in G2/M phase compared with controls (mean percentage of cells 10.7% control VS 15.3% RFWD3 ^Δ/Δ^ clone 3, p=0.013; mean percentage of cells 10.7% control VS 18.6% RFWD3 ^Δ/Δ^ clone 44, p=0.0001) (**Figure 4C**). This indicates that RFWD3 can modify cell cycle progression, either directly or indirectly through accumulation of DNA breaks due to ICL repair loss, and deficiency of RFWD3 results in G2/M checkpoint arrest.

Cell growth was measured over 72h (**Supplementary Figure S13**). Doubling times of RFWD3^Δ/Δ^ cells were significantly increased compared with controls (mean doubling time 16.1h control VS 20.8h RFWD3 ^Δ/Δ^ clone 3, p=0.028; mean doubling time 16.1h control VS 29.8h RFWD3 ^Δ/Δ^ clone 44, p<0.0001) (**Figure 4D**), demonstrating that deficiency of RFWD3 results in impairment of tumour cell proliferation. This again may be either a direct effect of RFWD3 loss, or an indirect effect resulting from accumulation of DNA damage.

Motility of cells in 2D was measured on collagen I and laminin substrates (**Supplementary Figure S14**), as these are major components of the ovarian extracellular matrix. Wild type and empty vector control ES2 cells were capable of migration on both substrates. In comparison, RFWD3^Δ/Δ^ cells showed significantly impaired migration velocities both on collagen I (mean velocity 1.28µm/min control VS 0.94µm/min RFWD3 ^Δ/Δ^ clone 3, p=0.001; mean velocity 1.28µm/min control VS 0.72µm/min RFWD3 ^Δ/Δ^ clone 44, p<0.0001) and laminin (mean velocity 0.99µm/min control VS 0.66µm/min RFWD3 ^Δ/Δ^ clone 3, p=0.014; mean velocity 0.99µm/min control VS 0.60µm/min RFWD3 ^Δ/Δ^ clone 44, p=0.005) (**Figures 4E, F**), indicating that RFWD3 can influence cell migration.

### 3.6 RFWD3 expression varies in a high grade serous ovarian cancer patient cohort and is associated with response to platinum therapy and progression free survival

To explore potential relationships between RFWD3 expression patterns at the protein level and outcomes in HGSOC, tumour microarrays were immunostained for RFWD3. Microarrays were generated consisting of 231 evaluable tumour cores sampled from a cohort of chemonaïve HGSOC patients at primary surgery. Summary clinicopathological information for this cohort is given in **Supplementary Tables S3** and **S4**.

**Figure 5:**
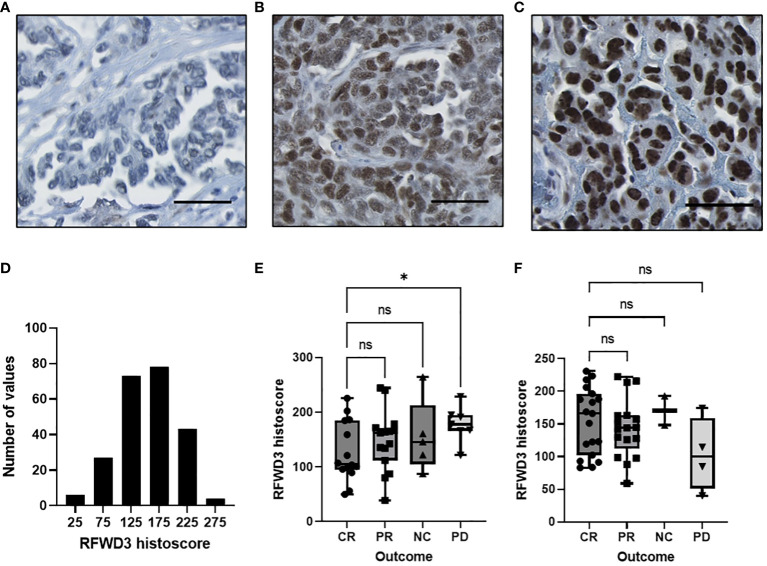
Expression of RFWD3 protein varies in a HGSOC cohort and is associated with response to platinum. **(A-C)** Representative images of RFWD3 staining in HGSOC tumour cores showing range of staining intensities in tumour nuclei. Brown staining shows RFWD3 expression, blue staining shows counterstain. Scale bars 100µm. **(D)** Histogram showing RFWD3 histoscores for tumour cores across the cohort. Bin size 50. **(E, F)** Box plots showing RFWD3 expression for tumours separated by response to chemotherapy. **(E)** cases treated with platinum based chemotherapy without taxanes and **(F)** cases treated with platinum and taxane based chemotherapy. CR, complete response; PR, partial response; NC, no change; PD, progressive disease by RECIST criteria. * p<0.05, ns, not significant via Welch’s ANOVA with Dunnett’s T3 multiple comparisons test. Box plots show 25^th^, 50^th^ and 75^th^ quartiles, whiskers extend to maximum and minimum points. Individual points shown.

RFWD3 staining predominantly occurred in cell nuclei, and intensity was highly varied across the cohort (**Figures 5A-C**). Histoscores, describing the overall proportion of strong, moderate, weak and negative staining, were generated for each core based on the nuclear staining intensity in tumour cells. These were normally distributed (**Figure 5D**), therefore the cases were split into “high” “mid” and “low” expression groups based on quartiles, with histoscores greater than Q3 assigned as “high” expression, less than Q1 assigned as “low” expression, and remaining cases assigned as “mid” level expression. Differences in clinical characteristics for the RFWD3 expression groups were assessed. No significant associations were found between RFWD3 expression and patient age, tumour stage, residual disease, or *BRCA1/2* mutation status (**Supplementary Table S3**).

Response to first line therapy was assessed for the tumours. Radiological response data was available for 83 of the tumours analysed for RFWD3 expression. A total of 42 of these were treated with platinum and taxanes, while 41 were treated with platinum without taxanes. As the number of cases with radiological response data available was low, quartile group data could not be analysed due to insufficient group numbers to give meaningful results. Therefore, response was assessed by comparing RFWD3 histoscores for each outcome for the different treatment types.

Significantly lower RFWD3 expression was observed for those patients with a complete response (CR) compared to those who showed progressive disease (PD) for the patients treated with platinum only (median histoscores 105 CR VS 178 PD, p=0.046) (**Figure 5E**). This supports a role for RFWD3 expression in tumour platinum response. In contrast, for those patients treated with platinum and taxane based therapy, there was no significant difference between those with a complete response to chemotherapy and those with progressive disease (**Figure 5F**).

The survival data was also analysed for this patient cohort, to determine whether the differences in therapeutic outcomes observed would translate into survival differences for patients with altered RFWD3 expression. Multivariate analyses of OS and PFS were carried out using the Cox proportional hazards model (**Table 1**). Age and residual disease were accounted for in the models, and the models were stratified by stage as this did not fit the proportional hazards assumption. Counter to expectations, the RFWD3 high-expression group had an improved PFS compared to the mid-expression group (hazard ratio 0.46-0.99, p=0.045). There was no significant difference in survival for the low-expression group. This finding however did not translate to a change in OS, as there were no significant changes for any of the RFWD3 expression groups.

**Table 1:** Cox proportional hazards analyses of PFS and OS for a HGSOC patient cohort.

		Hazard ratio	95% CI	p-value
**PFS**	RFWD3 high	0.68	0.46 - 0.99	0.045
	RFWD3 low	0.93	0.63 - 1.37	0.701
	Age	1.01	1.00 - 1.02	0.186
	Macroscopic disease after debulking surgery	0.86	0.56 - 1.33	0.503
**OS**	RFWD3 high	0.80	0.56 - 1.13	0.205
	RFWD3 low	0.94	0.65 - 1.35	0.725
	Age	1.02	1.00 - 1.03	0.015
	Macroscopic disease after debulking surgery	0.90	0.60 - 1.35	0.612

RFWD3 expression, age and macroscopic disease status following surgery were modelled, and PFS model was stratified by disease stage. CI confidence interval, PFS progression free survival, OS overall survival.

## 4 Discussion

Previous work has established that while mutations are uncommon to rare in HGSOC, excluding those which occur in *TP53* and *BRCA1/2*, HGSOC has a diverse genetic and molecular landscape (5, 28). We have found that despite the lack of mutations, RFWD3 expression is varied in HGSOC, and a subset of cases with low expression of RFWD3 were identified in the TCGA-OV dataset. Analyses of tissue samples and cell lines provided further evidence of this, with a wide range of RFWD3 protein staining observed in TMA samples, and limited RFWD3 expression observed in a HGSOC cell line. Reduced expression of other components of the FA pathway has been demonstrated to impair DNA damage repair capacity, conferring platinum sensitivity upon ovarian tumour cells and leading to favourable patient prognosis (31–33). We therefore investigated further the phenotypic and molecular impact of alterations in RFWD3 expression.

In the TCGA-OV dataset, cases with low RFWD3 expression were associated with increased tumour mutational burden and mutation count. This shows that changes in RFWD3 expression are sufficient to drive phenotypic changes in HGSOCs. In unstressed cells, RFWD3 has been proposed to maintain genomic stability by stabilizing DNA replication forks during normal replication processes (18). In the context of cancer cells in particular, which undergo rapid proliferation and are often subject to genotoxic stresses such as chemotherapy, the role of RFWD3 in the FA and HR pathways of DNA repair is also crucial for the maintenance of genomic stability (13, 30). Consistent with our work, accumulation of mutations has previously been observed in *RFWD3* mutated fibroblasts, and can be corrected by addition of wild type *RFWD3* (13). We build on this by providing evidence of a more definitive link between decreased RFWD3 expression and the acquisition of a genomic instability phenotype in tumours.

CRISPR-mediated gene editing was used to generate stable cell models of reduced RFWD3 expression. However, while hypomorphic mutations were introduced, we were unable to completely eliminate expression of RFWD3 from cells. This is consistent with the results of other groups, who have attempted to produce knockouts of RFWD3 using a wide variety of cell types and edit sites (15, 18, 30). Therefore, it may be the case that *RFWD3* is an essential gene for cell survival. Our group has successfully produced knockouts of another FA pathway gene using CRISPR-mediated gene editing (**Supplementary Figure S9**), and examples exist in the literature of the successful knockout of other FA pathway genes (34). Therefore, the essential nature of RFWD3 is likely unrelated to its role in FA mediated DNA repair, and may be a result of an alternative role of RFWD3, such as that which it plays in DNA replication (18).

RFWD3 depletion in HGSOC cell lines resulted in enhanced carboplatin sensitivity, both in transient and stable knockdown models. Previous studies have identified RFWD3 as a top hit for induction of cisplatin sensitivity in a genome wide siRNA screen (35), and shown that deficiency and mutation of RFWD3 are linked to cisplatin sensitivity (13, 15). Due to the shared mechanism of action of cisplatin and carboplatin (36), it is not unexpected that reduced RFWD3 expression also resulted in carboplatin sensitivity in the models that we used. Cisplatin has been shown to cause cell death via apoptosis in ovarian cancer cells (37, 38) which correlates with chemosensitivity in patient samples (39). This indicates that p53 and FAS ligand dependent apoptosis as a consequence of unrepaired ICLs (40) may be greatly enhanced by RFWD3 deficiency. Knockdown of RFWD3 may therefore restrict the ability of the cell to carry out ICL repair, leaving cells lacking RFWD3 vulnerable to platinum therapies. This could be exploited in cancers expressing lower levels of RFWD3.

Cells with reduced expression of RFWD3 also showed increased sensitivity to mitomycin C which, similar to platinum, induces ICLs in DNA (13). This confirmed that accumulation of ICLs is likely the cause of the enhanced sensitivity of RFWD3 deficient cells to carboplatin. While mitomycin C is not used routinely in the treatment of HGSOC, it reportedly may have clinical utility in *BRCA1* mutated ovarian cancers, particularly in heavily pre-treated and recurrent cancers with poor prognosis (41, 42). Mitomycin C also exhibits differing toxicity profiles to carboplatin (42), so could be used in combination with standard platinum chemotherapies, in cases with low expression of RFWD3. A preliminary study has demonstrated that this may be an effective approach in *BRCA1* mutated ovarian cancer (43).

In contrast to the enhanced sensitivity to platinum, no consistent change in paclitaxel sensitivity was observed in cell lines with reduced expression of RFWD3. This further demonstrates the value of using ICL inducing therapies over other options in the treatment of tumours with low RFWD3 expression.

The enhanced sensitivity of RFWD3 deficient cells to these DNA damaging agents is likely due to the key roles it fulfils in DNA damage repair, in particular ICL repair. RFWD3 is known to mediate ubiquitination of RPA at stalled replication forks to promote HR repair (17) and RAD51 to trigger unloading from DNA damage sites, and thus enable loading of late-stage HR factors and successful completion of HR (15). In the FA pathway it also functions to promote TLS by ubiquitination of single stranded DNA bound proteins, ultimately leading to stimulation of gap-filling DNA synthesis (14). As cell death in response to both mitomycin C and platinum-containing compounds is thought to be due to induction of ICLs (13, 36), this is likely the main cause of RFWD3 mediated sensitivity to these agents. Due to these multiple roles that RFWD3 plays in ICL repair, enhanced sensitivity to these agents may be a consequence of bulky adducts from unresolved ICLs blocking transcription and DNA replication, or accumulation of unresolved DSBs resulting from incomplete ICL repair or replication fork collapse (14, 15, 17). It is noted that the STRING database (https://string-db.org/) (44) also lists interactions of RFWD3 with PRIMPOL, MLKL and MDM2. PRIMPOL is involved in response to replication stress and stalled fork restart, and MDM2 is involved in the p53 mediated response to DNA damage response. Therefore, multiple roles of RFWD3 may also contribute to the observed platinum sensitivity (16, 45). Further investigation of DNA damage repair in RFWD3 deficient cells may help to elucidate a precise mechanism for the observed platinum and mitomycin C sensitivity.

The role of RFWD3 in platinum resistance was further demonstrated in an analysis of HGSOC patient tumour samples. A complete response to platinum-based chemotherapy was associated with lower expression of RFWD3 compared with progressive disease. However, this was not the case for those patients treated with platinum plus taxane combination therapy. This heightened sensitivity to platinum is consistent with the previous finding that RFWD3 deficient cells were hypersensitive to platinum therapy. It is unknown why those patients treated with both platinum and taxane failed to demonstrate the same low RFWD3 expression in complete responders as the group treated with platinum but not taxanes. A systematic review of the literature on platinum and taxane resistance suggests an inverse relationship between platinum and taxane resistance, i.e. cells that are resistant to carboplatin are more sensitive to taxanes (46). However, this is disputed, with some trials showing a survival benefit of platinum and taxane therapy compared to platinum alone in platinum sensitive ovarian cancer (47). Our data suggests that RFWD3 may have some utility as a biomarker of platinum sensitivity, and that patients with low RFWD3 expression may benefit more from treatment with platinum-based and ICL inducing chemotherapy, but not taxane-based therapies.

Another way in which RFWD3 may contribute to cellular phenotype is via its role in the cell cycle. We have shown here that in HGSOC cells, RFWD3 deficiency can lead to an accumulation of cells in G2/M phase, indicating a potential checkpoint block. This has also been previously reported in gastric cancer cells (21). A third group have reported a trend towards increased proportion of cells in G2/M phase in the U20S osteosarcoma cell line (18), however this only achieved statistical significance with one siRNA tested. However, they did observe an increased proportion of cells in S-phase (18). A possible explanation for this is the difference in genetic background between the cell lines. The ES2 cell line carries a *TP53* mutation, characteristic of HGSOC (5), which may result in dysregulation of the S-phase checkpoint regardless of RFWD3 expression status (48), whereas the U2OS cell line is *TP53* wild type. As platinum containing drugs also cause arrest in G2/M phase followed by apoptosis (49), cell cycle dysregulation may be an important contributor to the enhanced platinum sensitivity of cells with reduced RFWD3 expression.

We have also demonstrated further non-canonical roles of RFWD3 in HGSOC cells for the first time, including effects on cell proliferation and migration. A link between reduced RFWD3 expression and impaired cell proliferation has been previously shown in lung, colorectal and gastric cancers (20–22). This may be a result of the dysregulated cell cycle we observed in this cell line, with accumulation in G2/M phase leading to inhibition of cell growth. In gastric and colorectal cancer, RFWD3 was also associated with a cell migratory phenotype (21, 22). Interestingly, these factors are thought to be linked to a signalling role of RFWD3 separate from the roles it plays in DNA damage repair. Downregulation of RFWD3 has been associated with decreased activation of ERK, AKT and P38, and decreased expression of slug and N-cadherin, all of which are implicated in tumourigenesis (21). Direct interaction between RFWD3 and the E2F1 transcription factor has been shown to increase expression of BIRC5 and thus increase cell proliferation and migration (22). Consistent with this, targeting of BIRC5 in ovarian cancer has previously been found to reduce migration and invasion *in vitro*, and metastasis *in vivo* (50). In spite of this, similar to our findings for RFWD3, overexpression of BIRC5 has been associated with survival benefits in HGSOC (51). This indicates that the survival results that we observed may be related to the non-canonical signalling functions of RFWD3, and are not entirely unexpected.

Despite the evidence that low RFWD3 may convey a favourable cellular phenotype in terms of proliferation, migration, and chemosensitivity, no differences were observed in survival between the RFWD3 low-expressing tumours and RFWD3 mid-expressing tumours during analysis of a HGSOC patient cohort. Indeed, those tumours which expressed the highest levels of RFWD3 demonstrated the best survival outcomes. This may be reflective of the conflicting roles which RFWD3 plays in the tumour setting. One of the underlying characteristics of Fanconi anaemia is a predisposition to cancer due to increased levels of genomic instability (52), and BRCA1 and BRCA2 mutations are known to predispose individuals to breast and ovarian cancers (53, 54). We have demonstrated that low expression of RFWD3 also leads to increased frequency of mutations in HGSOC tumours. Conversely, high expression of RFWD3 may lead to a tumour with greater genomic stability, which may therefore accumulate fewer mutations, resulting in a lower degree of intratumour heterogeneity. The relationship between genomic instability, intratumour heterogeneity and prognosis is not straightforward, with pan-cancer and ovarian cancer studies suggesting that while some level of instability is favourable for cancer cell survival, beyond a certain point it may in fact be disadvantageous (55, 56). High intratumour heterogeneity has been associated with reduced progression-free survival in HGSOC, despite the fact that greater loss of heterozygosity is associated with good prognosis (57). While loss of FA pathway proteins BRCA1 and BRCA2 is associated with good prognosis (7), the multiple roles distinct from the FA pathway demonstrated for RFWD3 in our study and others highlight the fact that the larger picture of protein functionality should be considered, making survival interpretation more complex.

In conclusion, multiple roles have been identified for RFWD3 in HGSOC, as a result of both its function in ICL repair and its further non-canonical signalling roles. RFWD3 has been shown to mediate a wide range of tumour relevant functions, including genomic stability, response to ICL inducing chemotherapies, regulation of the cell cycle, proliferation and migration. However, the interplay between these roles is complex, and should be further studied. In future, RFWD3 may have utility as a biomarker of platinum sensitivity, or as a drug target for sensitizing tumours to platinum.

## Data availability statement

The original contributions presented in the study are included in the article/**Supplementary Material**. Further inquiries can be directed to the corresponding authors.

## Ethics statement

The studies involving humans were approved by Lothian NRS Bioresource Ethics Committee. The studies were conducted in accordance with the local legislation and institutional requirements. The ethics committee/institutional review board waived the requirement of written informed consent for participation from the participants or the participants’ legal guardians/next of kin because the retrospective nature of the study.

## Author contributions

ST: Conceptualization, Formal analysis, Investigation, Methodology, Visualization, Writing – original draft, Writing – review & editing. RH: Methodology, Resources, Writing – review & editing. CG: Methodology, Resources, Writing – review & editing. CH: Methodology, Resources, Writing – review & editing. SL: Conceptualization, Funding acquisition, Supervision, Writing – review & editing. MA: Conceptualization, Funding acquisition, Supervision, Writing – review & editing.
