# Supplementary material for: RFWD3 modulates response to platinum chemotherapy and promotes cancer associated phenotypes in high grade serous ovarian cancer
Source: Front Oncol. 2024 Apr 22;14:1389472. doi: 10.3389/fonc.2024.1389472 (PMC11071161; doi:10.3389/fonc.2024.1389472)
Supplement: Supplementary file 1 [file Presentation_1.pptx]

## Slide 1
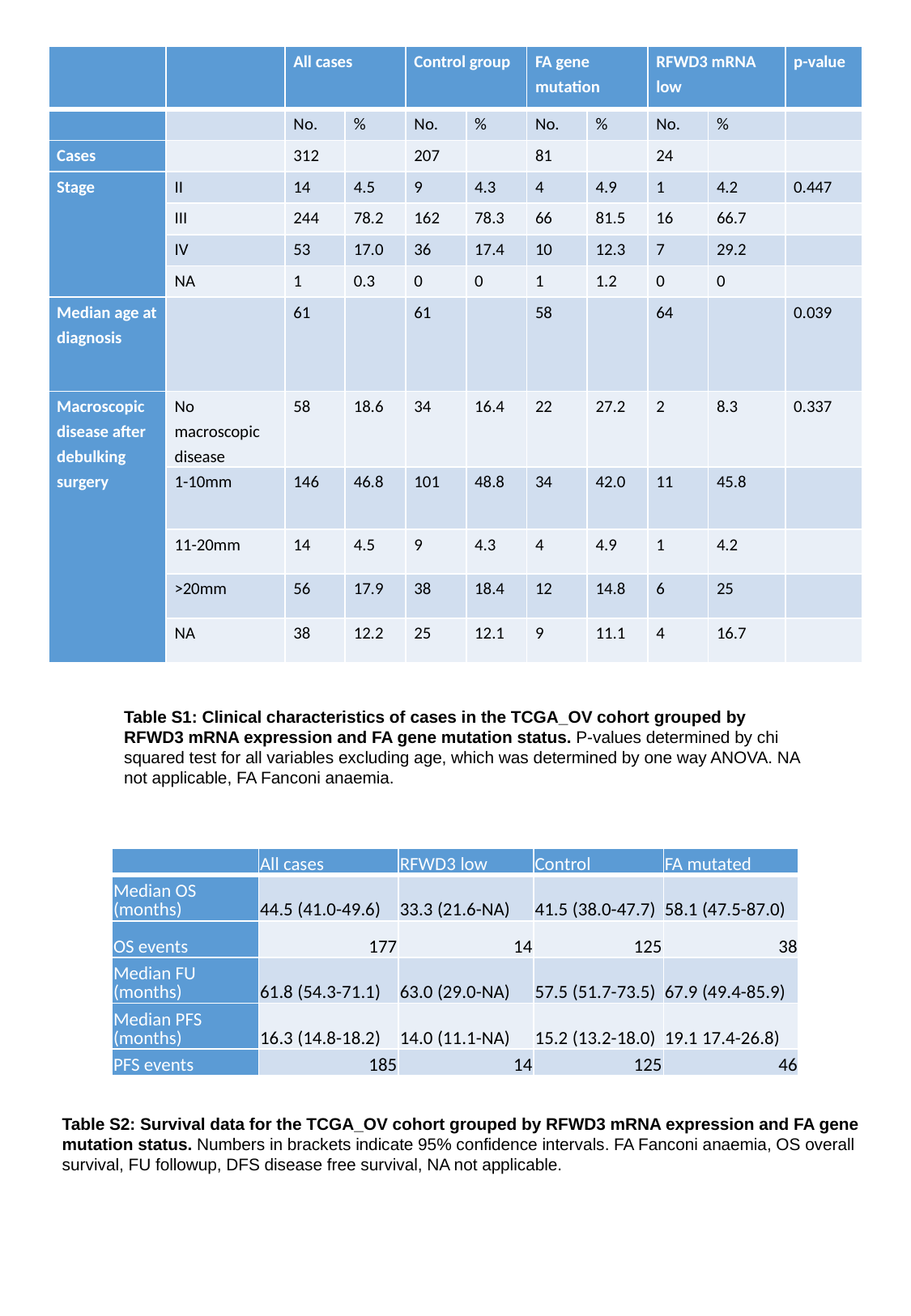

| | | All cases | | Control group | | FA gene mutation | | RFWD3 mRNA low | | p-value |
| --- | --- | --- | --- | --- | --- | --- | --- | --- | --- | --- |
| | | No. | % | No. | % | No. | % | No. | % | |
| Cases | | 312 | | 207 | | 81 | | 24 | | |
| Stage | II | 14 | 4.5 | 9 | 4.3 | 4 | 4.9 | 1 | 4.2 | 0.447 |
| | III | 244 | 78.2 | 162 | 78.3 | 66 | 81.5 | 16 | 66.7 | |
| | IV | 53 | 17.0 | 36 | 17.4 | 10 | 12.3 | 7 | 29.2 | |
| | NA | 1 | 0.3 | 0 | 0 | 1 | 1.2 | 0 | 0 | |
| Median age at diagnosis | | 61 | | 61 | | 58 | | 64 | | 0.039 |
| Macroscopic disease after debulking surgery | No macroscopic disease | 58 | 18.6 | 34 | 16.4 | 22 | 27.2 | 2 | 8.3 | 0.337 |
| | 1-10mm | 146 | 46.8 | 101 | 48.8 | 34 | 42.0 | 11 | 45.8 | |
| | 11-20mm | 14 | 4.5 | 9 | 4.3 | 4 | 4.9 | 1 | 4.2 | |
| | >20mm | 56 | 17.9 | 38 | 18.4 | 12 | 14.8 | 6 | 25 | |
| | NA | 38 | 12.2 | 25 | 12.1 | 9 | 11.1 | 4 | 16.7 | |
Table S1: Clinical characteristics of cases in the TCGA_OV cohort grouped by RFWD3 mRNA expression and FA gene mutation status. P-values determined by chi squared test for all variables excluding age, which was determined by one way ANOVA. NA not applicable, FA Fanconi anaemia.
| | All cases | RFWD3 low | Control | FA mutated |
| --- | --- | --- | --- | --- |
| Median OS (months) | 44.5 (41.0-49.6) | 33.3 (21.6-NA) | 41.5 (38.0-47.7) | 58.1 (47.5-87.0) |
| OS events | 177 | 14 | 125 | 38 |
| Median FU (months) | 61.8 (54.3-71.1) | 63.0 (29.0-NA) | 57.5 (51.7-73.5) | 67.9 (49.4-85.9) |
| Median PFS (months) | 16.3 (14.8-18.2) | 14.0 (11.1-NA) | 15.2 (13.2-18.0) | 19.1 17.4-26.8) |
| PFS events | 185 | 14 | 125 | 46 |
Table S2: Survival data for the TCGA_OV cohort grouped by RFWD3 mRNA expression and FA gene mutation status. Numbers in brackets indicate 95% confidence intervals. FA Fanconi anaemia, OS overall survival, FU followup, DFS disease free survival, NA not applicable.

## Slide 2
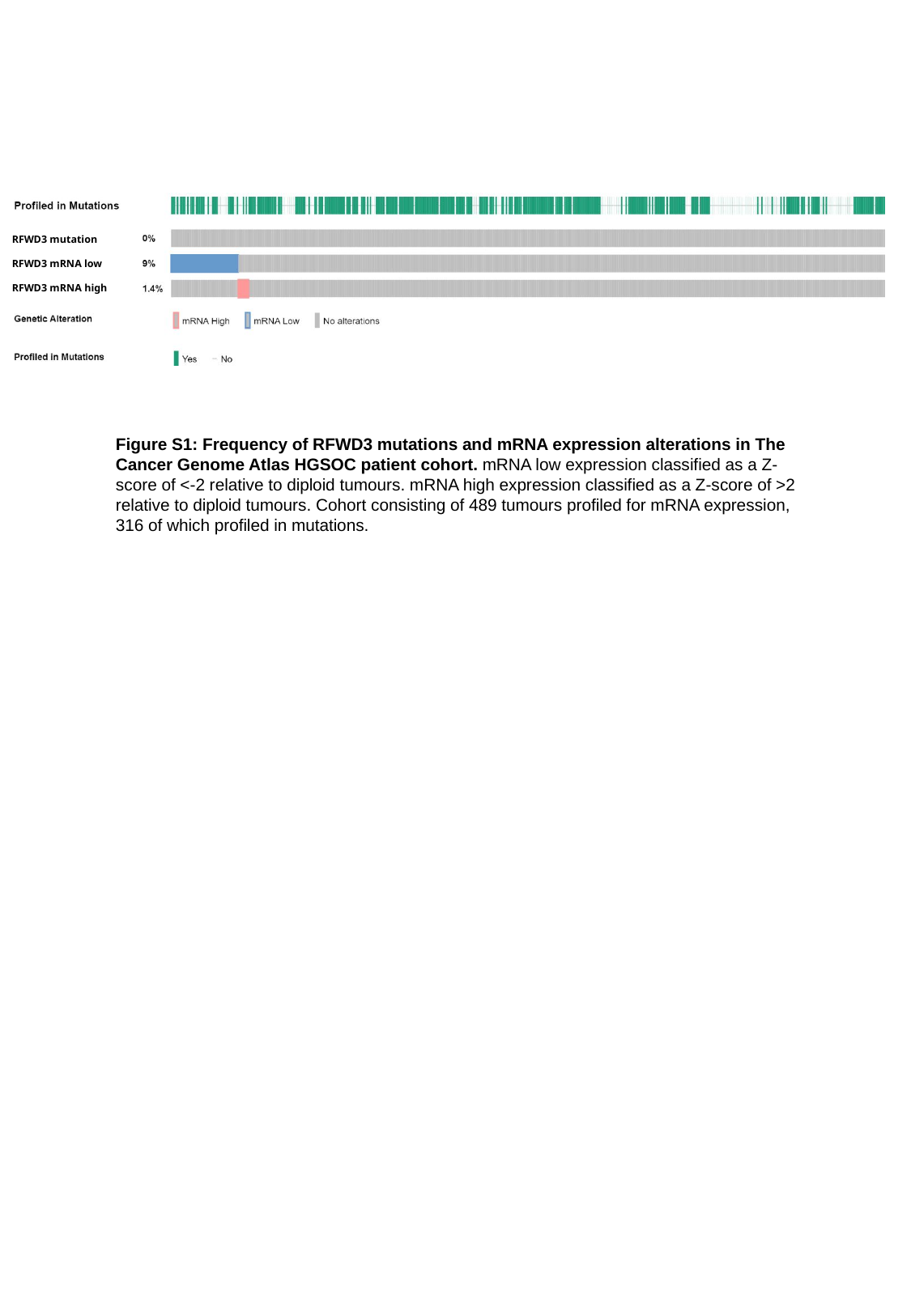

RFWD3 mutation
RFWD3 mRNA low
RFWD3 mRNA high
Figure S1: Frequency of RFWD3 mutations and mRNA expression alterations in The Cancer Genome Atlas HGSOC patient cohort. mRNA low expression classified as a Z-score of <-2 relative to diploid tumours. mRNA high expression classified as a Z-score of >2 relative to diploid tumours. Cohort consisting of 489 tumours profiled for mRNA expression, 316 of which profiled in mutations.

## Slide 3
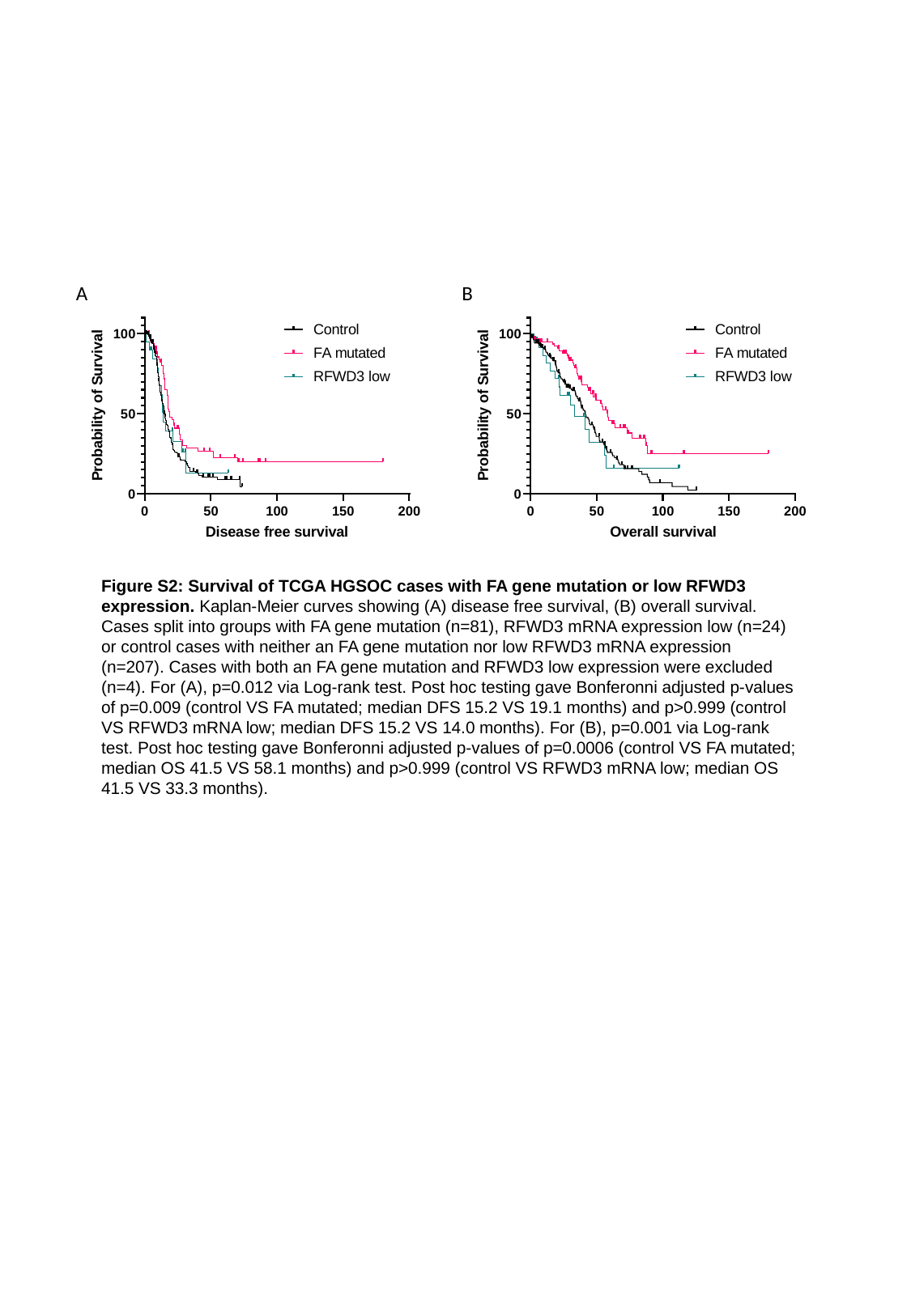

A
B
Figure S2: Survival of TCGA HGSOC cases with FA gene mutation or low RFWD3 expression. Kaplan-Meier curves showing (A) disease free survival, (B) overall survival. Cases split into groups with FA gene mutation (n=81), RFWD3 mRNA expression low (n=24) or control cases with neither an FA gene mutation nor low RFWD3 mRNA expression (n=207). Cases with both an FA gene mutation and RFWD3 low expression were excluded (n=4). For (A), p=0.012 via Log-rank test. Post hoc testing gave Bonferonni adjusted p-values of p=0.009 (control VS FA mutated; median DFS 15.2 VS 19.1 months) and p>0.999 (control VS RFWD3 mRNA low; median DFS 15.2 VS 14.0 months). For (B), p=0.001 via Log-rank test. Post hoc testing gave Bonferonni adjusted p-values of p=0.0006 (control VS FA mutated; median OS 41.5 VS 58.1 months) and p>0.999 (control VS RFWD3 mRNA low; median OS 41.5 VS 33.3 months).

## Slide 4
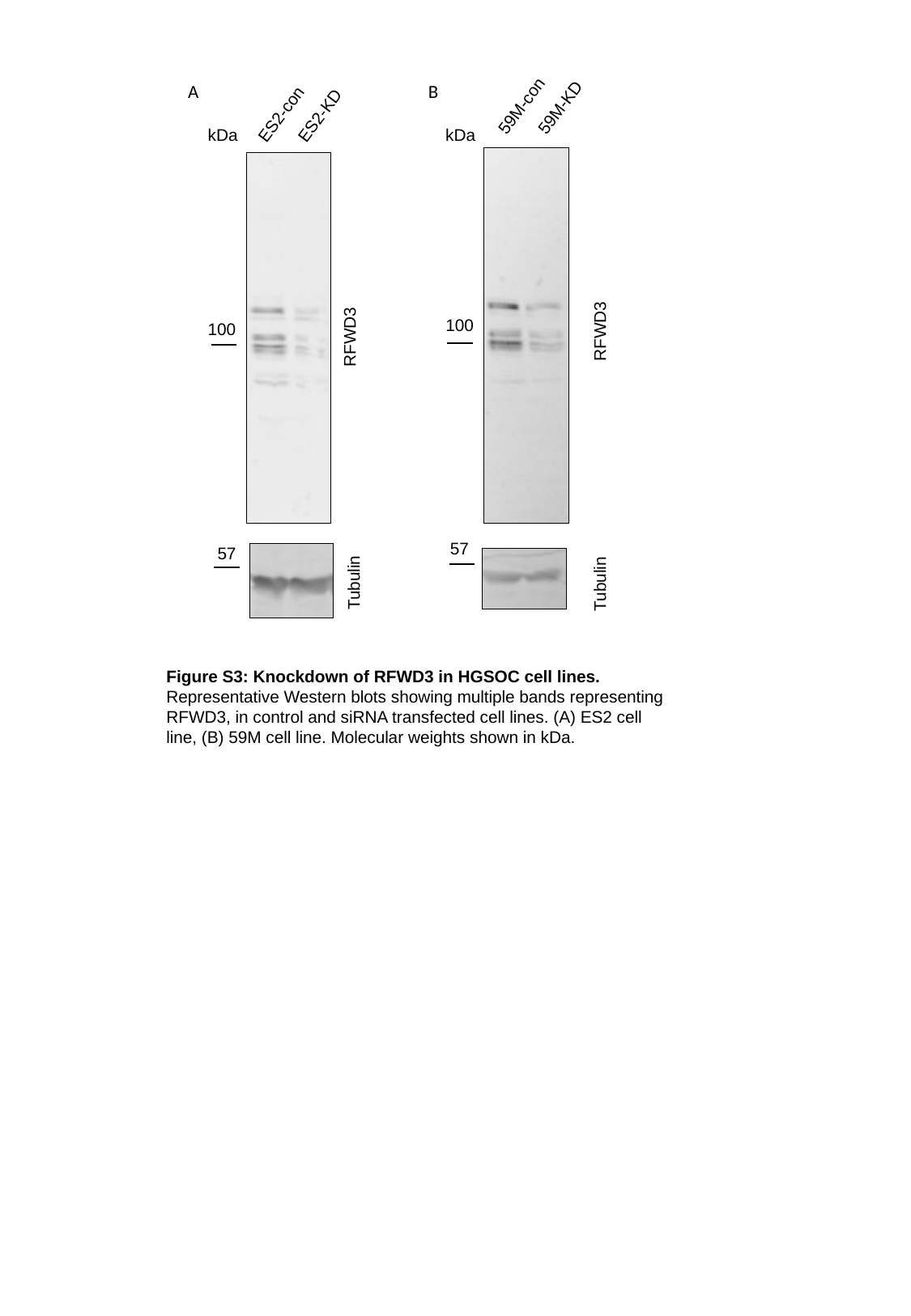

59M-con
59M-KD
100
RFWD3
ES2-con
ES2-KD
100
RFWD3
kDa
A
B
kDa
Tubulin
57
Tubulin
57
Figure S3: Knockdown of RFWD3 in HGSOC cell lines. Representative Western blots showing multiple bands representing RFWD3, in control and siRNA transfected cell lines. (A) ES2 cell line, (B) 59M cell line. Molecular weights shown in kDa.

## Slide 5
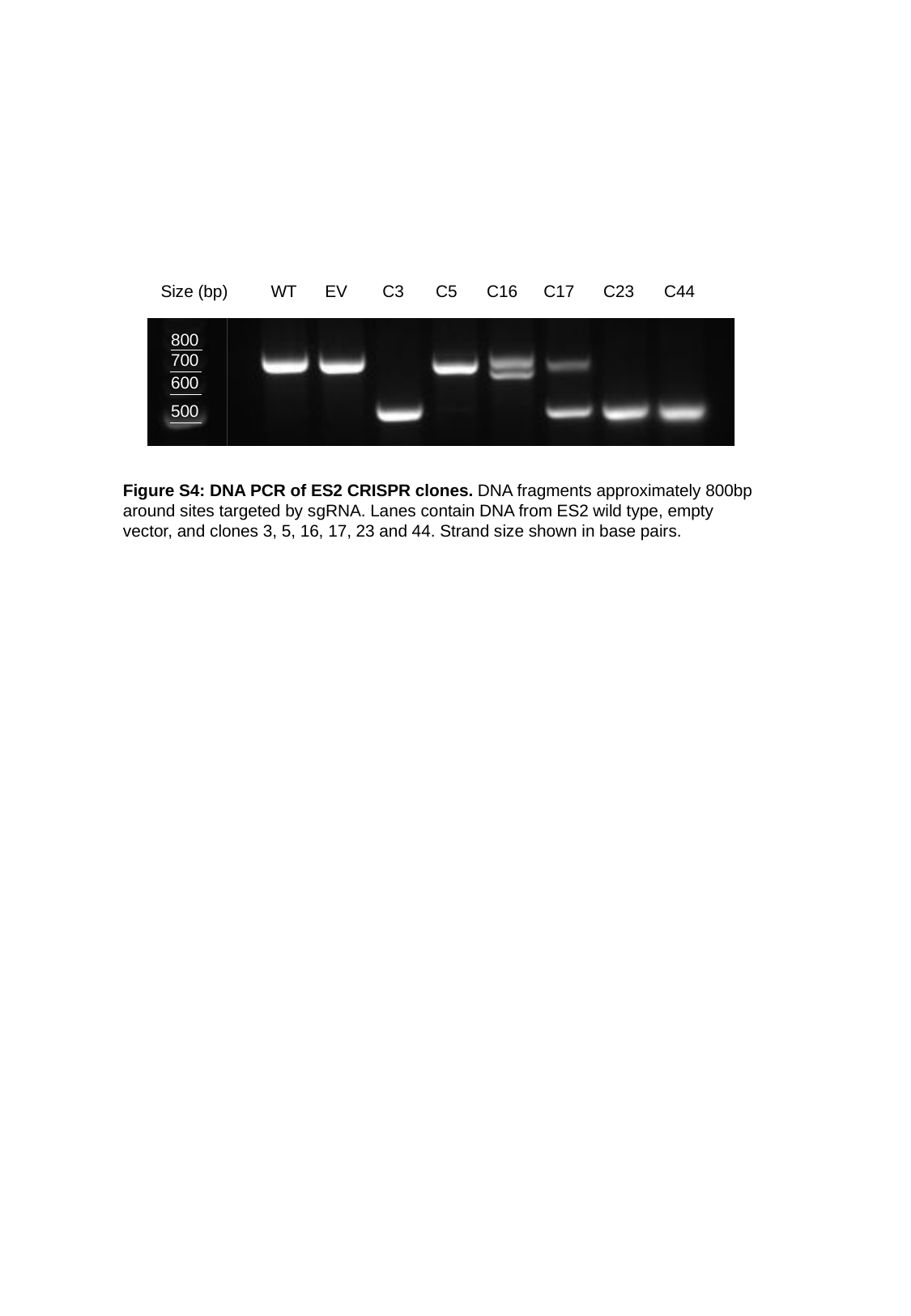

Size (bp)
WT
EV
C3
C5
C16
C17
C23
C44
800
700
600
500
Figure S4: DNA PCR of ES2 CRISPR clones. DNA fragments approximately 800bp around sites targeted by sgRNA. Lanes contain DNA from ES2 wild type, empty vector, and clones 3, 5, 16, 17, 23 and 44. Strand size shown in base pairs.

## Slide 6
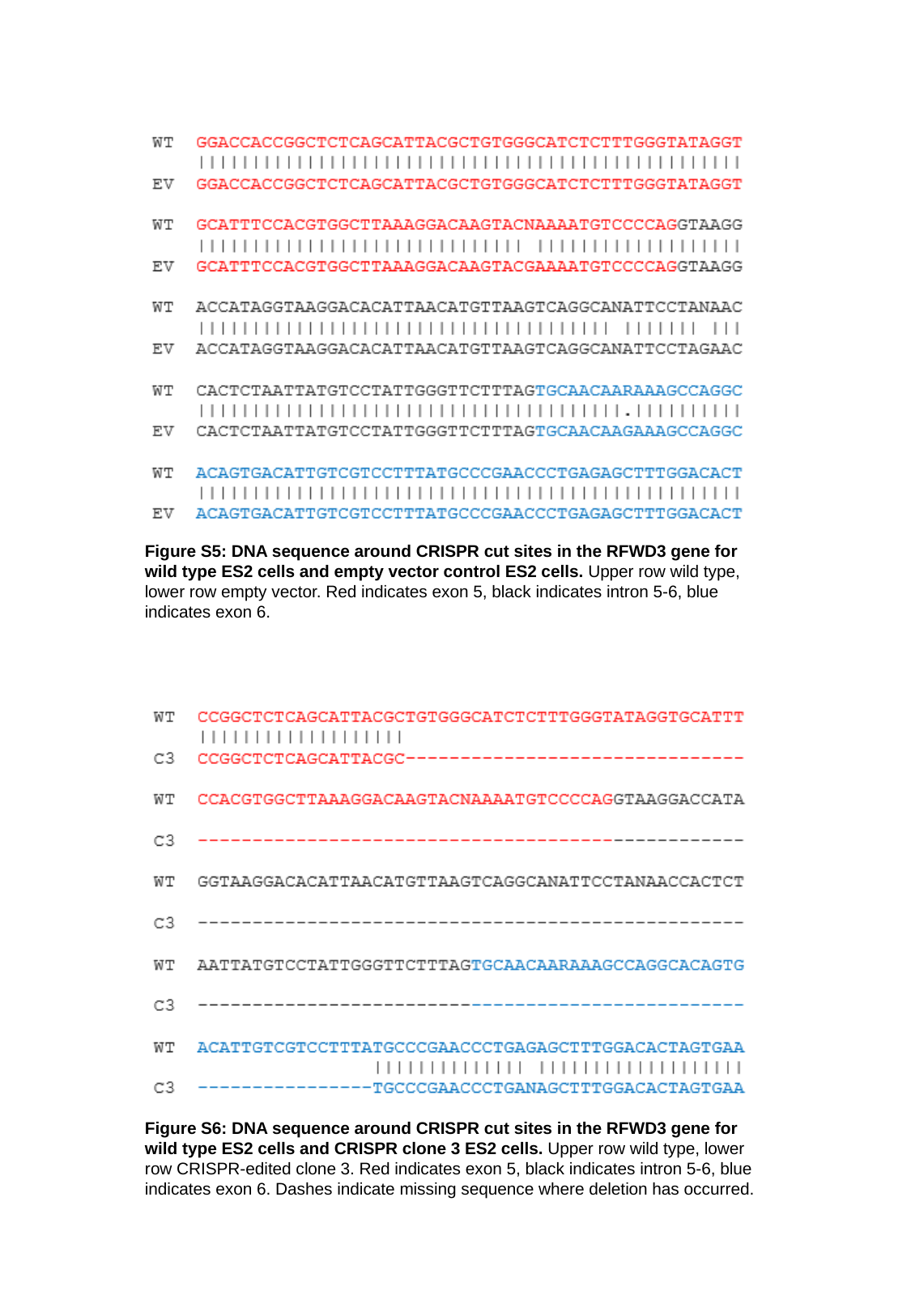

Figure S5: DNA sequence around CRISPR cut sites in the RFWD3 gene for wild type ES2 cells and empty vector control ES2 cells. Upper row wild type, lower row empty vector. Red indicates exon 5, black indicates intron 5-6, blue indicates exon 6.
Figure S6: DNA sequence around CRISPR cut sites in the RFWD3 gene for wild type ES2 cells and CRISPR clone 3 ES2 cells. Upper row wild type, lower row CRISPR-edited clone 3. Red indicates exon 5, black indicates intron 5-6, blue indicates exon 6. Dashes indicate missing sequence where deletion has occurred.

## Slide 7
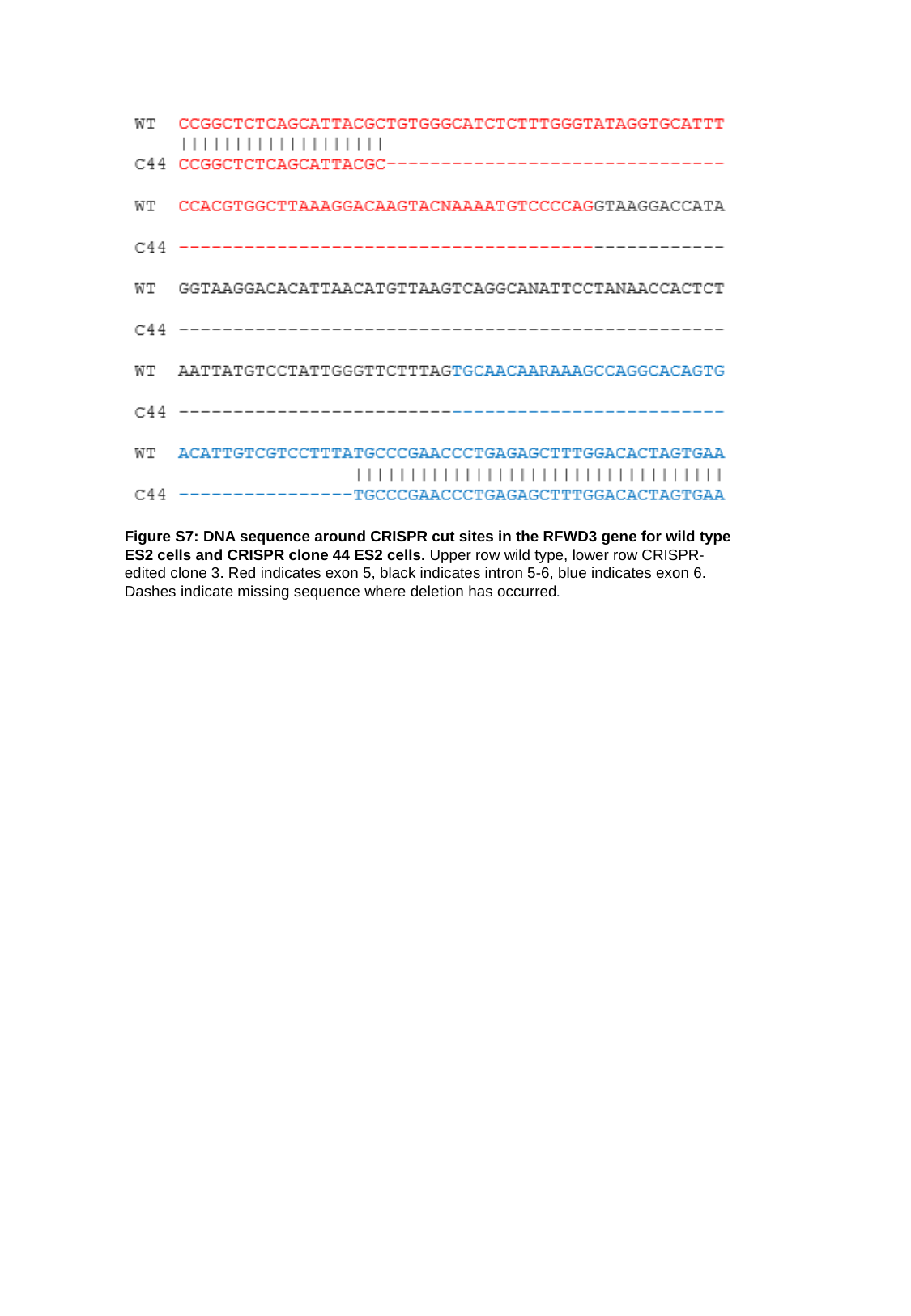

Figure S7: DNA sequence around CRISPR cut sites in the RFWD3 gene for wild type ES2 cells and CRISPR clone 44 ES2 cells. Upper row wild type, lower row CRISPR-edited clone 3. Red indicates exon 5, black indicates intron 5-6, blue indicates exon 6. Dashes indicate missing sequence where deletion has occurred.

## Slide 8
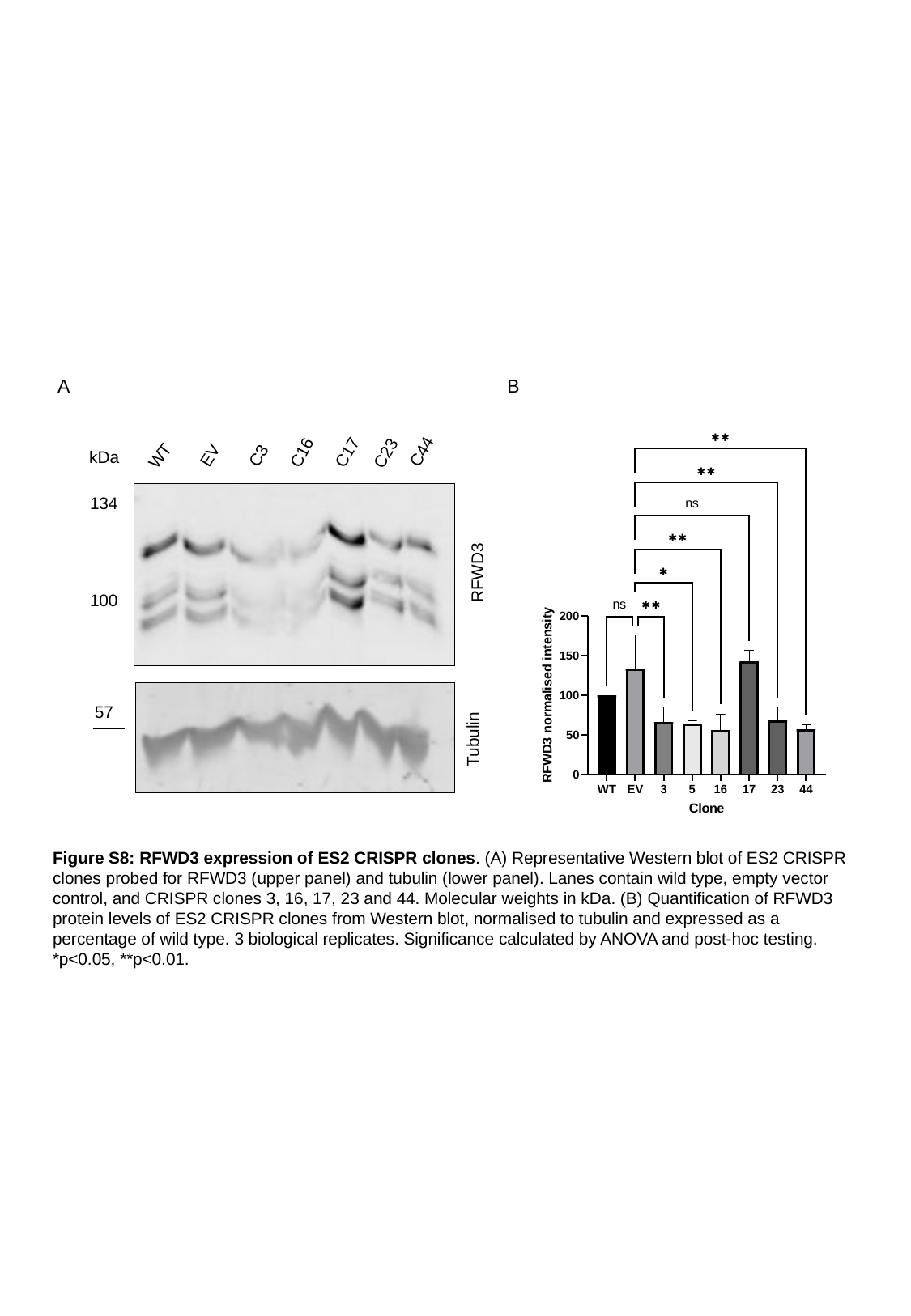

A
B
C44
C16
C17
C23
EV
C3
WT
kDa
134
RFWD3
100
Tubulin
57
Figure S8: RFWD3 expression of ES2 CRISPR clones. (A) Representative Western blot of ES2 CRISPR clones probed for RFWD3 (upper panel) and tubulin (lower panel). Lanes contain wild type, empty vector control, and CRISPR clones 3, 16, 17, 23 and 44. Molecular weights in kDa. (B) Quantification of RFWD3 protein levels of ES2 CRISPR clones from Western blot, normalised to tubulin and expressed as a percentage of wild type. 3 biological replicates. Significance calculated by ANOVA and post-hoc testing. *p<0.05, **p<0.01.

## Slide 9
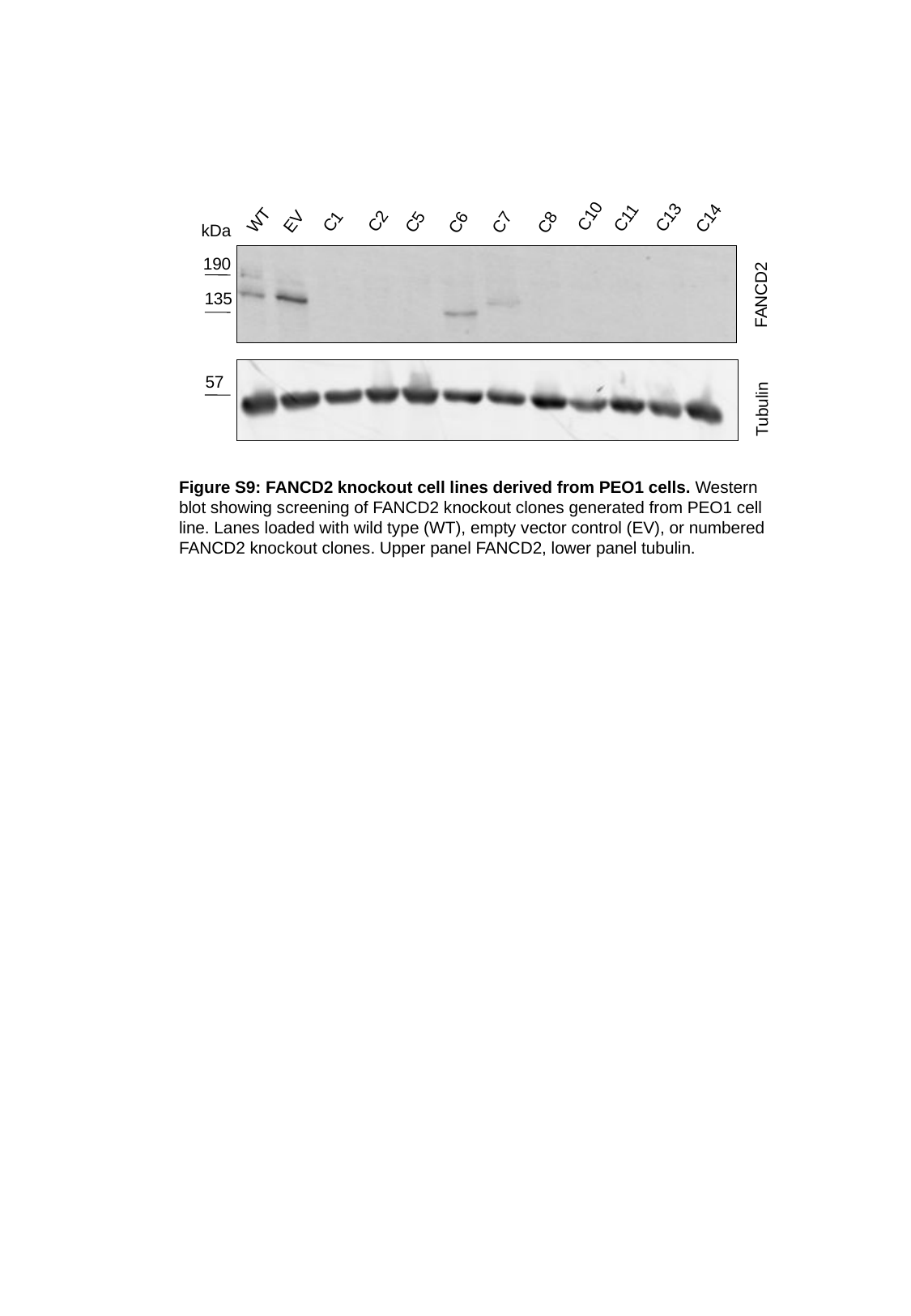

C10
C11
C13
C14
C2
C1
C5
EV
WT
C6
C7
C8
kDa
190
FANCD2
135
57
Tubulin
Figure S9: FANCD2 knockout cell lines derived from PEO1 cells. Western blot showing screening of FANCD2 knockout clones generated from PEO1 cell line. Lanes loaded with wild type (WT), empty vector control (EV), or numbered FANCD2 knockout clones. Upper panel FANCD2, lower panel tubulin.

## Slide 10
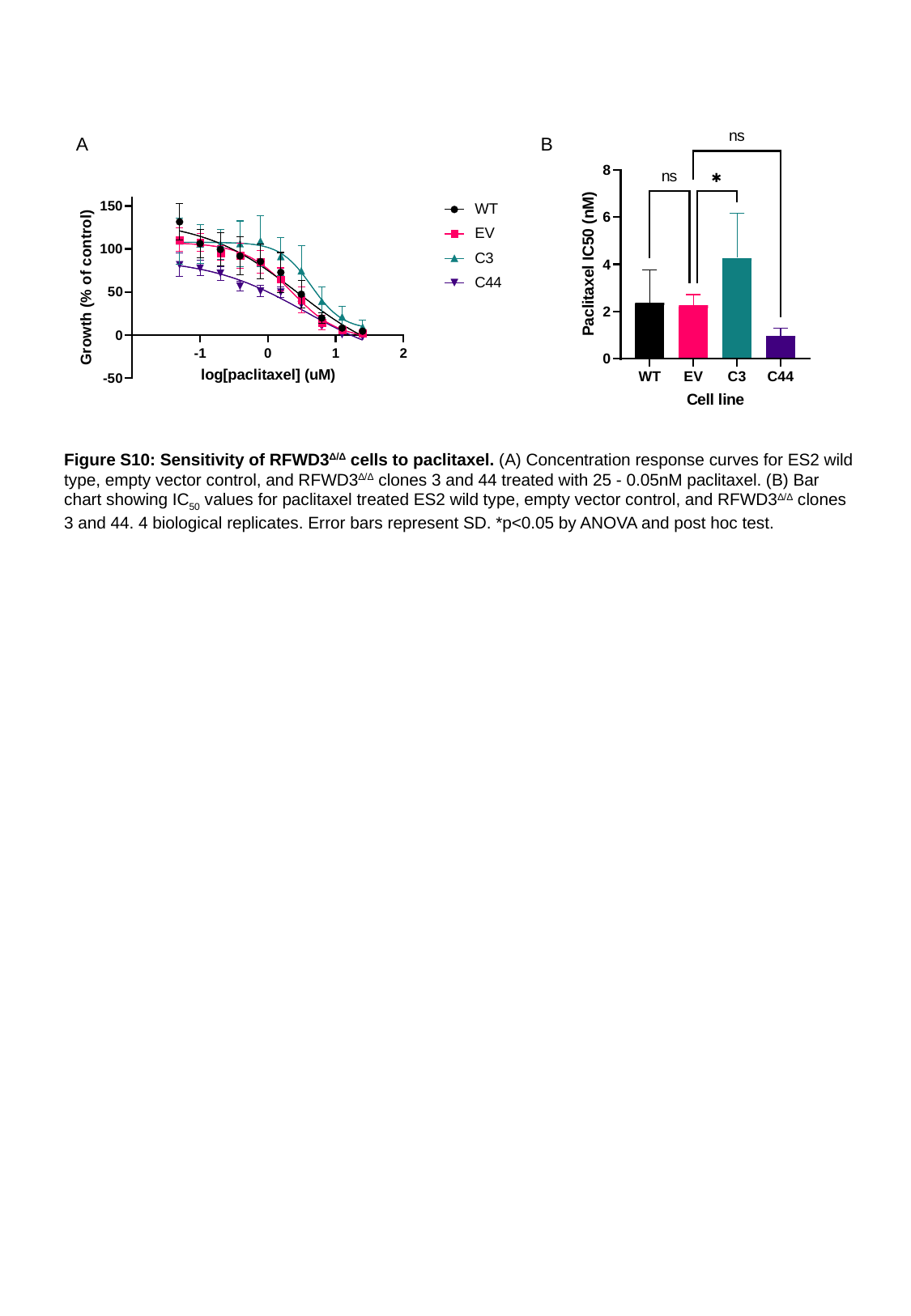

A
B
Figure S10: Sensitivity of RFWD3Δ/Δ cells to paclitaxel. (A) Concentration response curves for ES2 wild type, empty vector control, and RFWD3Δ/Δ clones 3 and 44 treated with 25 - 0.05nM paclitaxel. (B) Bar chart showing IC50 values for paclitaxel treated ES2 wild type, empty vector control, and RFWD3Δ/Δ clones 3 and 44. 4 biological replicates. Error bars represent SD. *p<0.05 by ANOVA and post hoc test.

## Slide 11
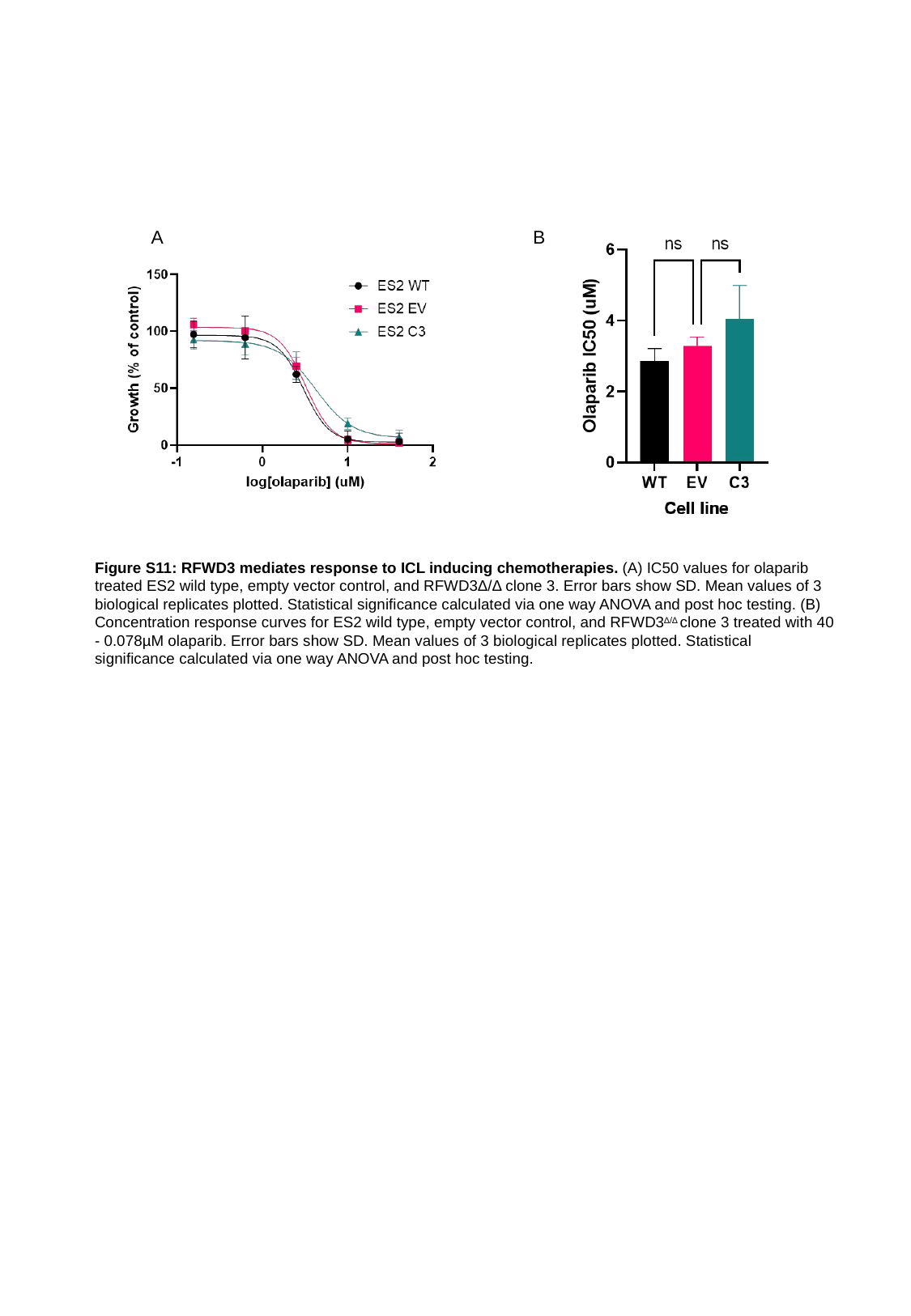

A
B
Figure S11: RFWD3 mediates response to ICL inducing chemotherapies. (A) IC50 values for olaparib treated ES2 wild type, empty vector control, and RFWD3Δ/Δ clone 3. Error bars show SD. Mean values of 3 biological replicates plotted. Statistical significance calculated via one way ANOVA and post hoc testing. (B) Concentration response curves for ES2 wild type, empty vector control, and RFWD3Δ/Δ clone 3 treated with 40 - 0.078µM olaparib. Error bars show SD. Mean values of 3 biological replicates plotted. Statistical significance calculated via one way ANOVA and post hoc testing.

## Slide 12
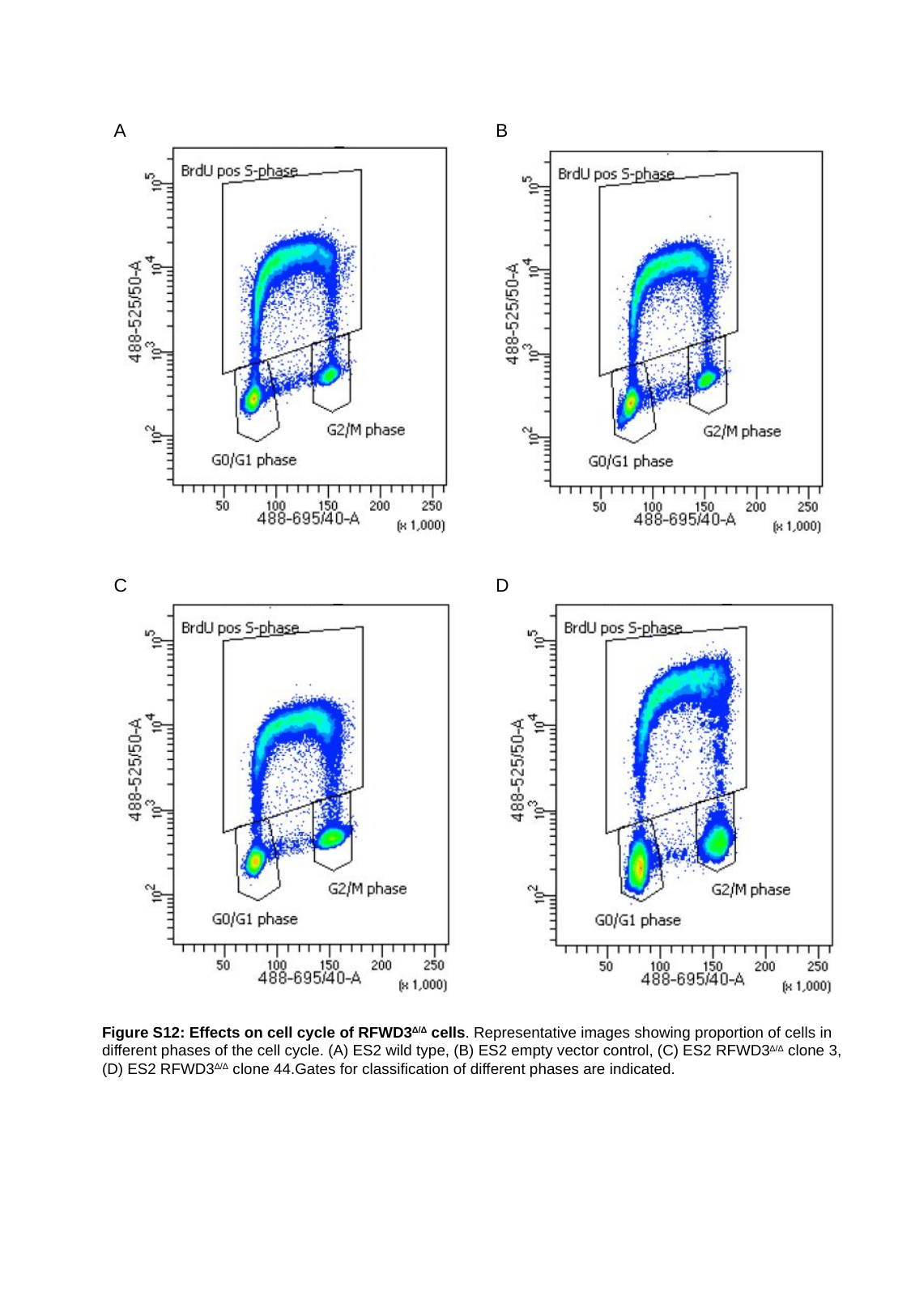

A
B
C
D
Figure S12: Effects on cell cycle of RFWD3Δ/Δ cells. Representative images showing proportion of cells in different phases of the cell cycle. (A) ES2 wild type, (B) ES2 empty vector control, (C) ES2 RFWD3Δ/Δ clone 3, (D) ES2 RFWD3Δ/Δ clone 44.Gates for classification of different phases are indicated.

## Slide 13
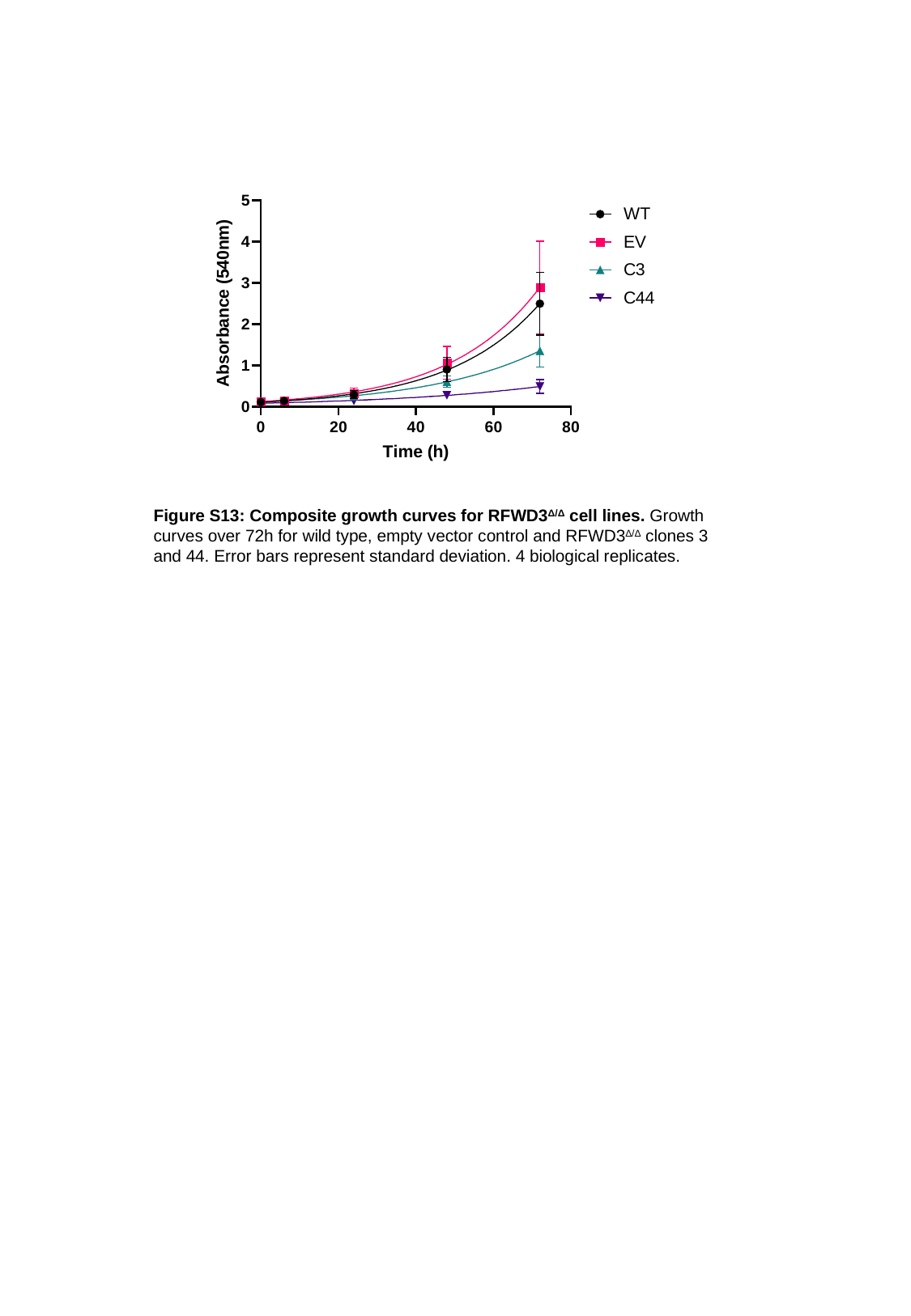

Figure S13: Composite growth curves for RFWD3Δ/Δ cell lines. Growth curves over 72h for wild type, empty vector control and RFWD3Δ/Δ clones 3 and 44. Error bars represent standard deviation. 4 biological replicates.

## Slide 14
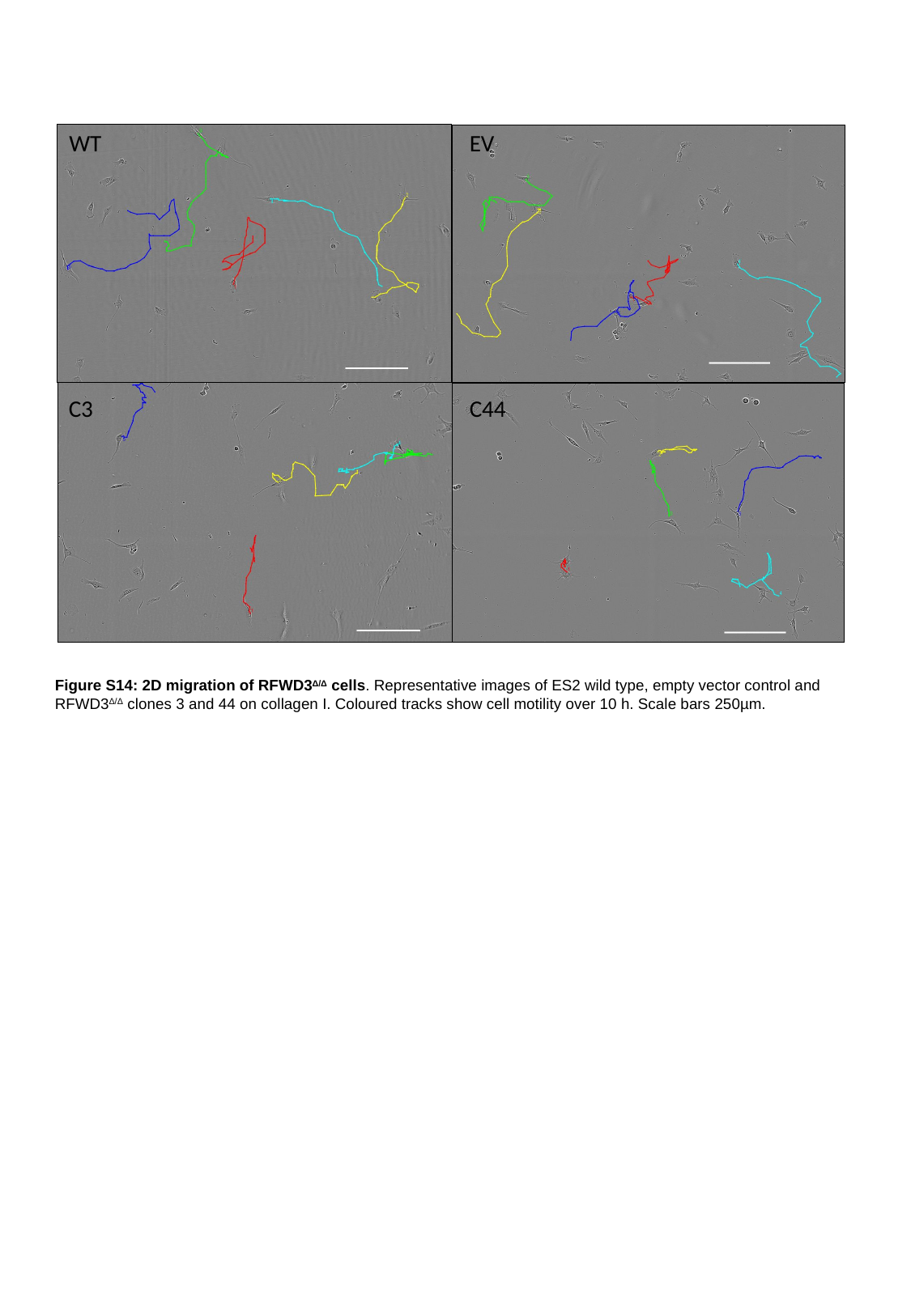

WT
EV
C3
C44
Figure S14: 2D migration of RFWD3Δ/Δ cells. Representative images of ES2 wild type, empty vector control and RFWD3Δ/Δ clones 3 and 44 on collagen I. Coloured tracks show cell motility over 10 h. Scale bars 250µm.

## Slide 15
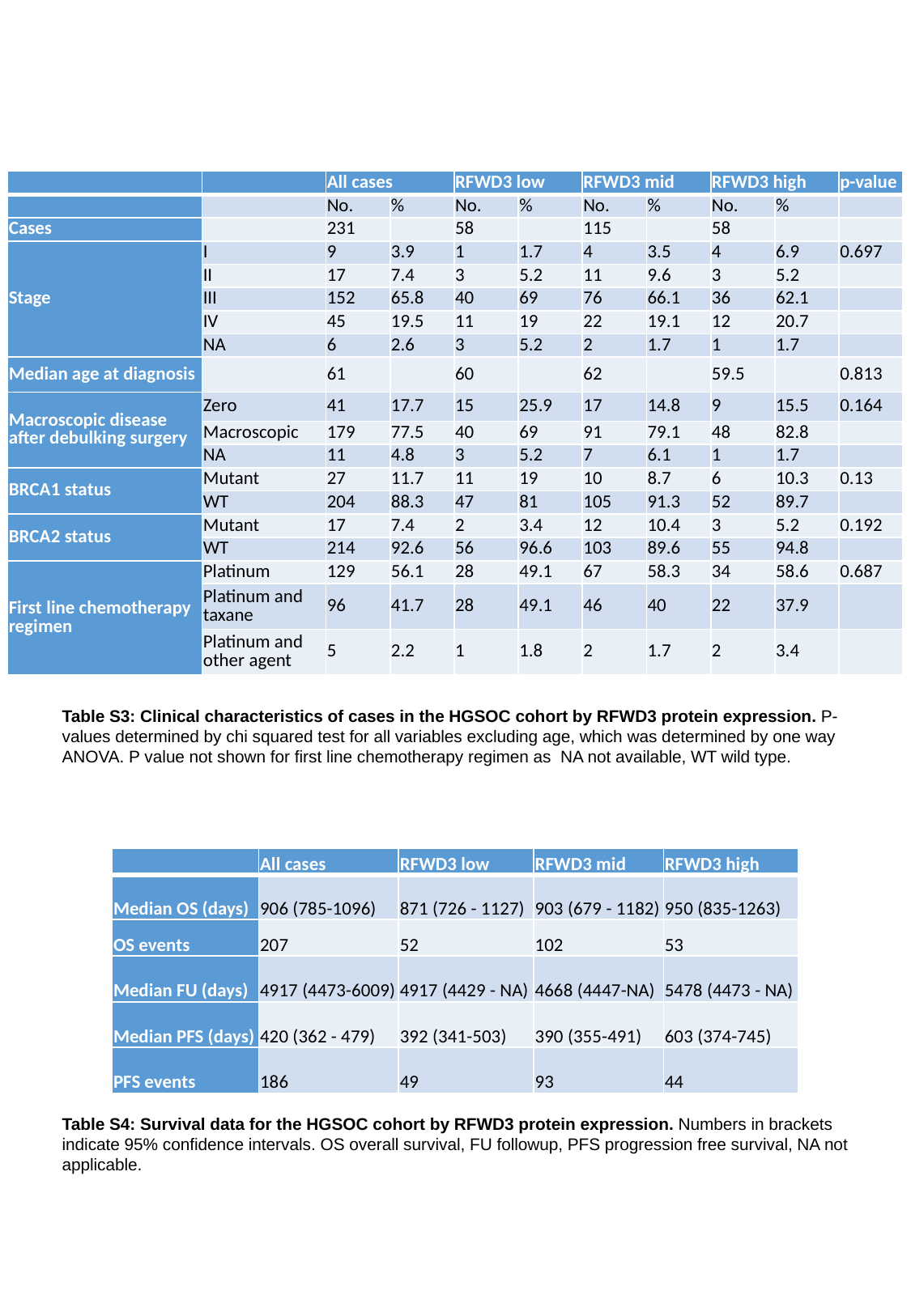

| | | All cases | | RFWD3 low | | RFWD3 mid | | RFWD3 high | | p-value |
| --- | --- | --- | --- | --- | --- | --- | --- | --- | --- | --- |
| | | No. | % | No. | % | No. | % | No. | % | |
| Cases | | 231 | | 58 | | 115 | | 58 | | |
| Stage | I | 9 | 3.9 | 1 | 1.7 | 4 | 3.5 | 4 | 6.9 | 0.697 |
| | II | 17 | 7.4 | 3 | 5.2 | 11 | 9.6 | 3 | 5.2 | |
| | III | 152 | 65.8 | 40 | 69 | 76 | 66.1 | 36 | 62.1 | |
| | IV | 45 | 19.5 | 11 | 19 | 22 | 19.1 | 12 | 20.7 | |
| | NA | 6 | 2.6 | 3 | 5.2 | 2 | 1.7 | 1 | 1.7 | |
| Median age at diagnosis | | 61 | | 60 | | 62 | | 59.5 | | 0.813 |
| Macroscopic disease after debulking surgery | Zero | 41 | 17.7 | 15 | 25.9 | 17 | 14.8 | 9 | 15.5 | 0.164 |
| | Macroscopic | 179 | 77.5 | 40 | 69 | 91 | 79.1 | 48 | 82.8 | |
| | NA | 11 | 4.8 | 3 | 5.2 | 7 | 6.1 | 1 | 1.7 | |
| BRCA1 status | Mutant | 27 | 11.7 | 11 | 19 | 10 | 8.7 | 6 | 10.3 | 0.13 |
| | WT | 204 | 88.3 | 47 | 81 | 105 | 91.3 | 52 | 89.7 | |
| BRCA2 status | Mutant | 17 | 7.4 | 2 | 3.4 | 12 | 10.4 | 3 | 5.2 | 0.192 |
| | WT | 214 | 92.6 | 56 | 96.6 | 103 | 89.6 | 55 | 94.8 | |
| First line chemotherapy regimen | Platinum | 129 | 56.1 | 28 | 49.1 | 67 | 58.3 | 34 | 58.6 | 0.687 |
| | Platinum and taxane | 96 | 41.7 | 28 | 49.1 | 46 | 40 | 22 | 37.9 | |
| | Platinum and other agent | 5 | 2.2 | 1 | 1.8 | 2 | 1.7 | 2 | 3.4 | |
Table S3: Clinical characteristics of cases in the HGSOC cohort by RFWD3 protein expression. P-values determined by chi squared test for all variables excluding age, which was determined by one way ANOVA. P value not shown for first line chemotherapy regimen as NA not available, WT wild type.
| | All cases | RFWD3 low | RFWD3 mid | RFWD3 high |
| --- | --- | --- | --- | --- |
| Median OS (days) | 906 (785-1096) | 871 (726 - 1127) | 903 (679 - 1182) | 950 (835-1263) |
| OS events | 207 | 52 | 102 | 53 |
| Median FU (days) | 4917 (4473-6009) | 4917 (4429 - NA) | 4668 (4447-NA) | 5478 (4473 - NA) |
| Median PFS (days) | 420 (362 - 479) | 392 (341-503) | 390 (355-491) | 603 (374-745) |
| PFS events | 186 | 49 | 93 | 44 |
Table S4: Survival data for the HGSOC cohort by RFWD3 protein expression. Numbers in brackets indicate 95% confidence intervals. OS overall survival, FU followup, PFS progression free survival, NA not applicable.

## Slide 16
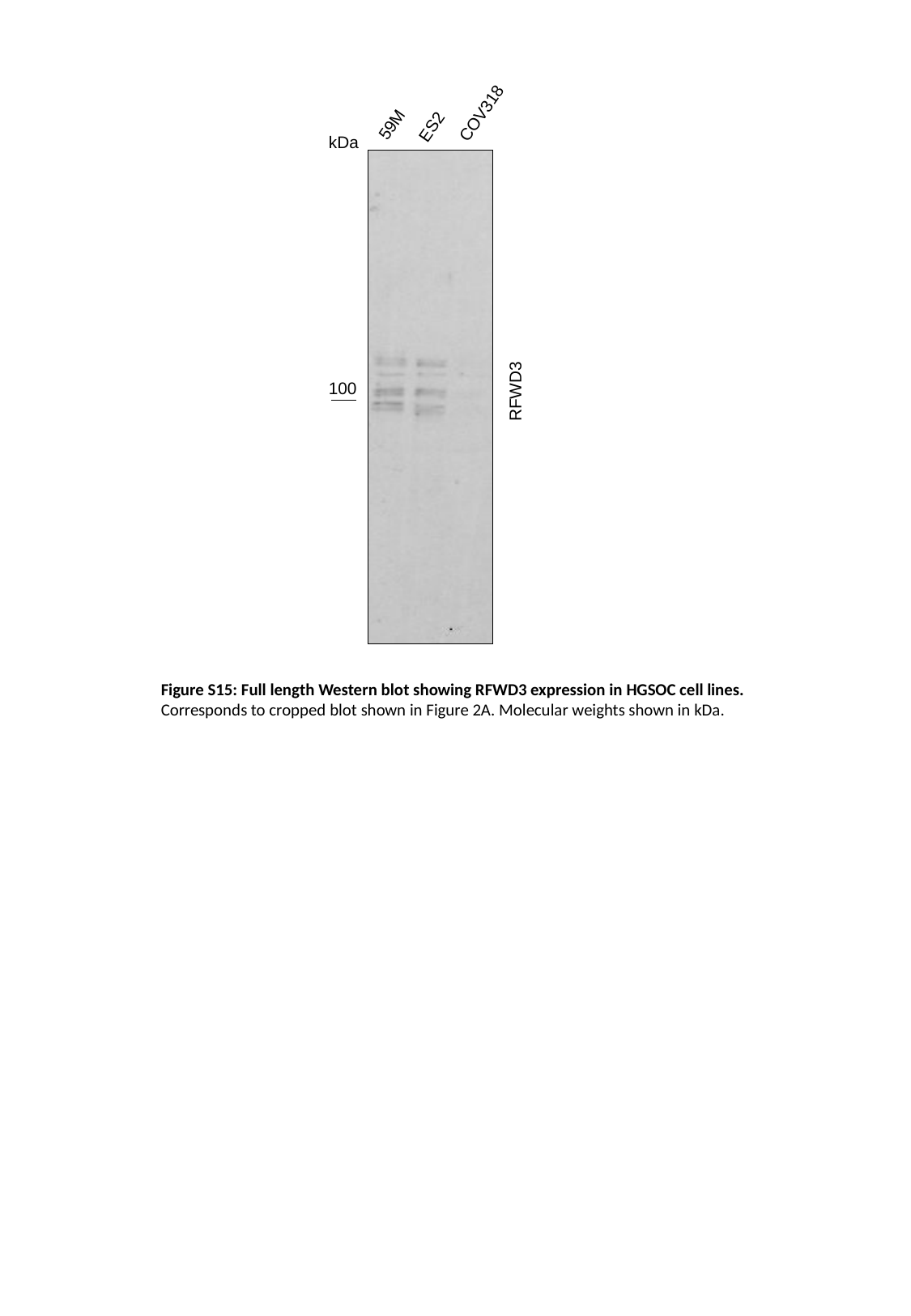

COV318
59M
ES2
kDa
RFWD3
100
Figure S15: Full length Western blot showing RFWD3 expression in HGSOC cell lines. Corresponds to cropped blot shown in Figure 2A. Molecular weights shown in kDa.

## Slide 17
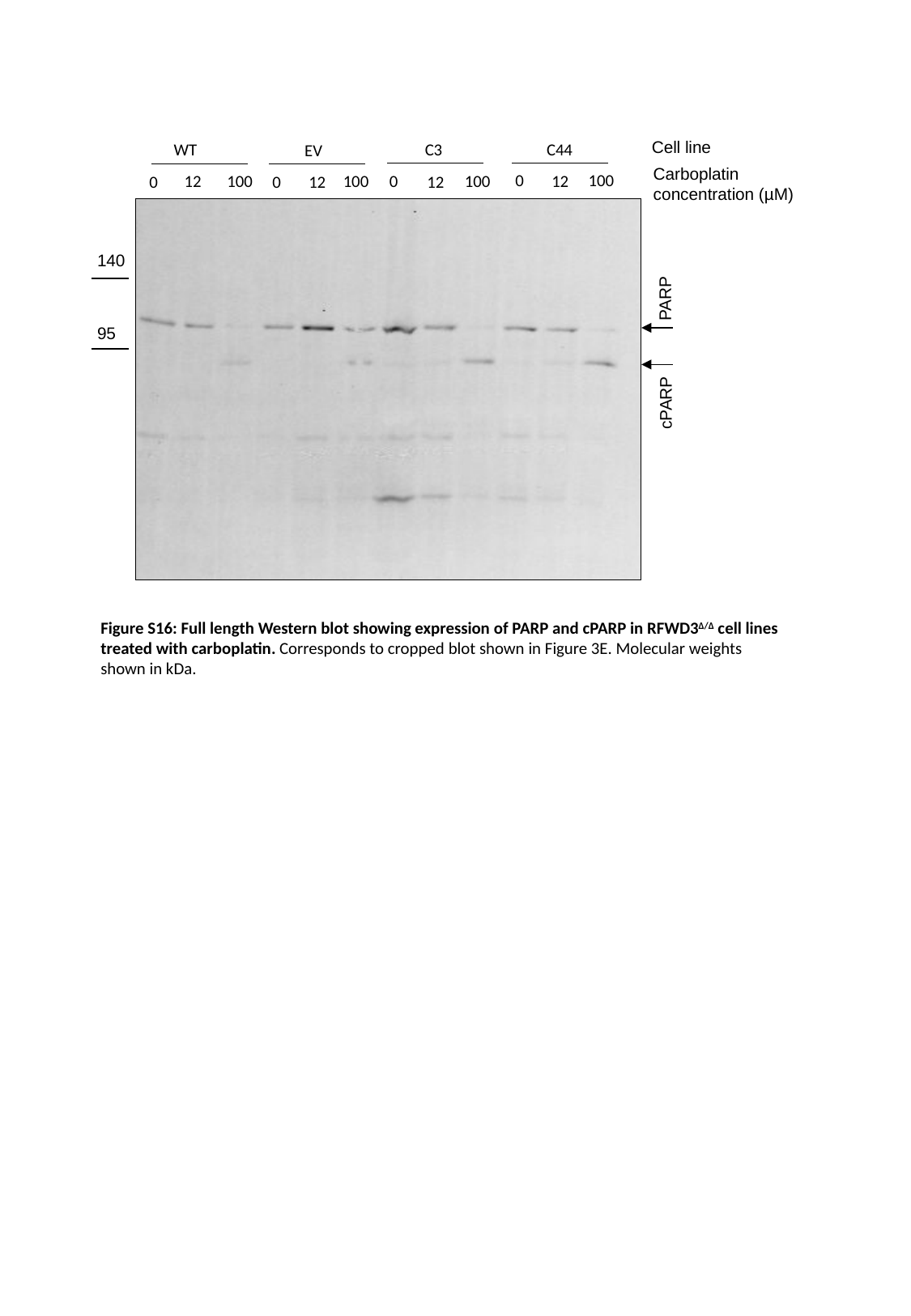

Cell line
C3
WT
C44
EV
0
100
100
100
12
0
100
12
0
12
0
12
140
PARP
95
cPARP
Carboplatin concentration (µM)
Figure S16: Full length Western blot showing expression of PARP and cPARP in RFWD3Δ/Δ cell lines treated with carboplatin. Corresponds to cropped blot shown in Figure 3E. Molecular weights shown in kDa.
